# Human endogenous retrovirus onco-exaptation counters cancer cell senescence through calbindin

**DOI:** 10.1172/JCI164397

**Published:** 2023-07-17

**Authors:** Jan Attig, Judith Pape, Laura Doglio, Anastasiya Kazachenka, Eleonora Ottina, George R. Young, Katey S.S. Enfield, Iker Valle Aramburu, Kevin W. Ng, Nikhil Faulkner, William Bolland, Venizelos Papayannopoulos, Charles Swanton, George Kassiotis

**Affiliations:** 1 Retroviral Immunology,; 2 Bioinformatics and Biostatistics,; 3 Cancer Evolution and Genome Instability, and; 4 Antimicrobial Defence, The Francis Crick Institute, London, United Kingdom.; 5 Department of Infectious Disease, Faculty of Medicine, Imperial College London, London, United Kingdom.

**Keywords:** Genetics, Oncology, Cellular senescence, Epigenetics, Lung cancer

## Abstract

Increased levels and diversity of human endogenous retrovirus (HERV) transcription characterize most cancer types and are linked with disease outcomes. However, the underlying processes are incompletely understood. Here, we show that elevated transcription of *HERVH* proviruses predicted survival of lung squamous cell carcinoma (LUSC) and identified an isoform of *CALB1*, encoding calbindin, ectopically driven by an upstream *HERVH* provirus under the control of KLF5, as the mediator of this effect. *HERVH*-*CALB1* expression was initiated in preinvasive lesions and associated with their progression. Calbindin loss in LUSC cell lines impaired in vitro and in vivo growth and triggered senescence, consistent with a protumor effect. However, calbindin also directly controlled the senescence-associated secretory phenotype (SASP), marked by secretion of CXCL8 and other neutrophil chemoattractants. In established carcinomas, *CALB1*-negative cancer cells became the dominant source of CXCL8, correlating with neutrophil infiltration and worse prognosis. Thus, *HERVH*-*CALB1* expression in LUSC may display antagonistic pleiotropy, whereby the benefits of escaping senescence early during cancer initiation and clonal competition were offset by the prevention of SASP and protumor inflammation at later stages.

## Graphical abstract

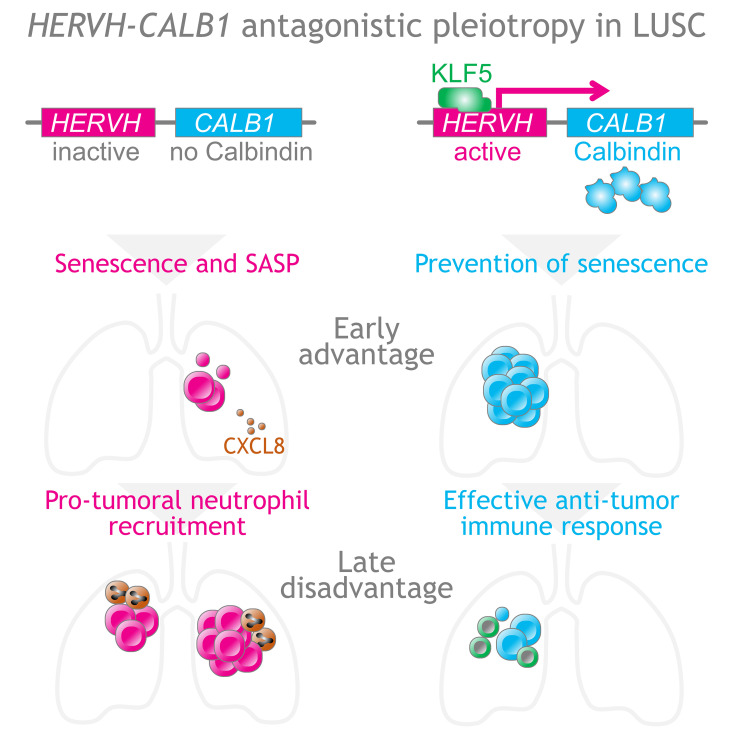

## Introduction

Tumor evolution follows distinguishable trajectories depending on the balance of multiple cancer cell–intrinsic and –extrinsic mechanisms working in concert or antagonistically. Protumor or antitumor effects arise from cancer cell–intrinsic processes regulating proliferation or senescence (1–3). Similarly, extrinsic factors, most notably distinct types of immune infiltrates, may either suppress or promote tumor growth (4–6). While traditionally investigated separately, cancer cell–intrinsic and –extrinsic processes may be connected by mechanisms that are incompletely understood. For example, the immune landscape of tumors depends on cancer cell–intrinsic genetic programs or oncogenic pathways (7–9). Also, cancer cell senescence shapes the tumor immune microenvironment through the senescence-associated secretory phenotype (SASP) (3). Therefore, cancer cell–intrinsic processes influence antitumor immunity, which in turn may affect tumor growth and progression through alternative evolution paths.

Despite their importance, the precise mechanisms or signals of bidirectional communication between cancer cells and antitumor immunity are only now beginning to emerge. One suggested mechanism incriminates endogenous retroelements (EREs), the cancer cell–intrinsic transcriptional reactivation of which has been linked with tumor immunogenicity (10, 11). Over 4 million ERE integrations are recognized in the human genome (12, 13), the vast majority being incomplete and mutated copies. Nevertheless, members of diverse ERE families can affect host physiology or induce pathology through retrotransposition-independent mechanisms (10, 14, 15). These include induction of an interferon response through production of nucleic acid ligands for innate immune DNA and RNA sensors (10, 11). Indeed, ERE-derived nucleic acids are considered the triggers of innate immunity in cancer as well as in autoimmune diseases and age-related inflammation (10, 11, 16–18). EREs may also affect cancer initiation and progression through onco-exaptation, whereby distinct integrations modify the function of neighboring genes or adopt new functions (19, 20). The overexpression of proto-oncogenes (21, 22) and of alternative oncogenic forms of kinases (23, 24) represent prime onco-exaptation examples.

Although growing, the list of onco-exaptation events is likely an underestimation of ERE potential, owing to lack of complete annotation of ERE transcriptional patterns, particularly in the complex epigenetic landscape and transcriptional dysregulation of cancer (22, 25). Employing de novo cancer transcriptome assembly, we have previously reported extensive transcriptional utilization of EREs defined by long-terminal repeats (LTRs) flanking the proviral genomes, which include human endogenous retroviruses (HERVs) and mammalian apparent LTR-retrotransposons (MaLRs) (25). Distinct cancer types express distinct LTR element–utilizing transcripts, suggesting cancer type–specific regulation as well as overall effect. Here, we examine the potential consequences for LTR element transcriptional activation in lung squamous cell carcinoma (LUSC) and identify a *HERVH*-driven isoform of *CALB1*, the gene encoding the calcium-binding protein calbindin, as a major determinant of cancer cell senescence, protumor inflammation, and patient survival.

## Results

### Cancer-specific HERVH-driven expression of CALB1 predicts LUSC survival.

**Figure 1:**
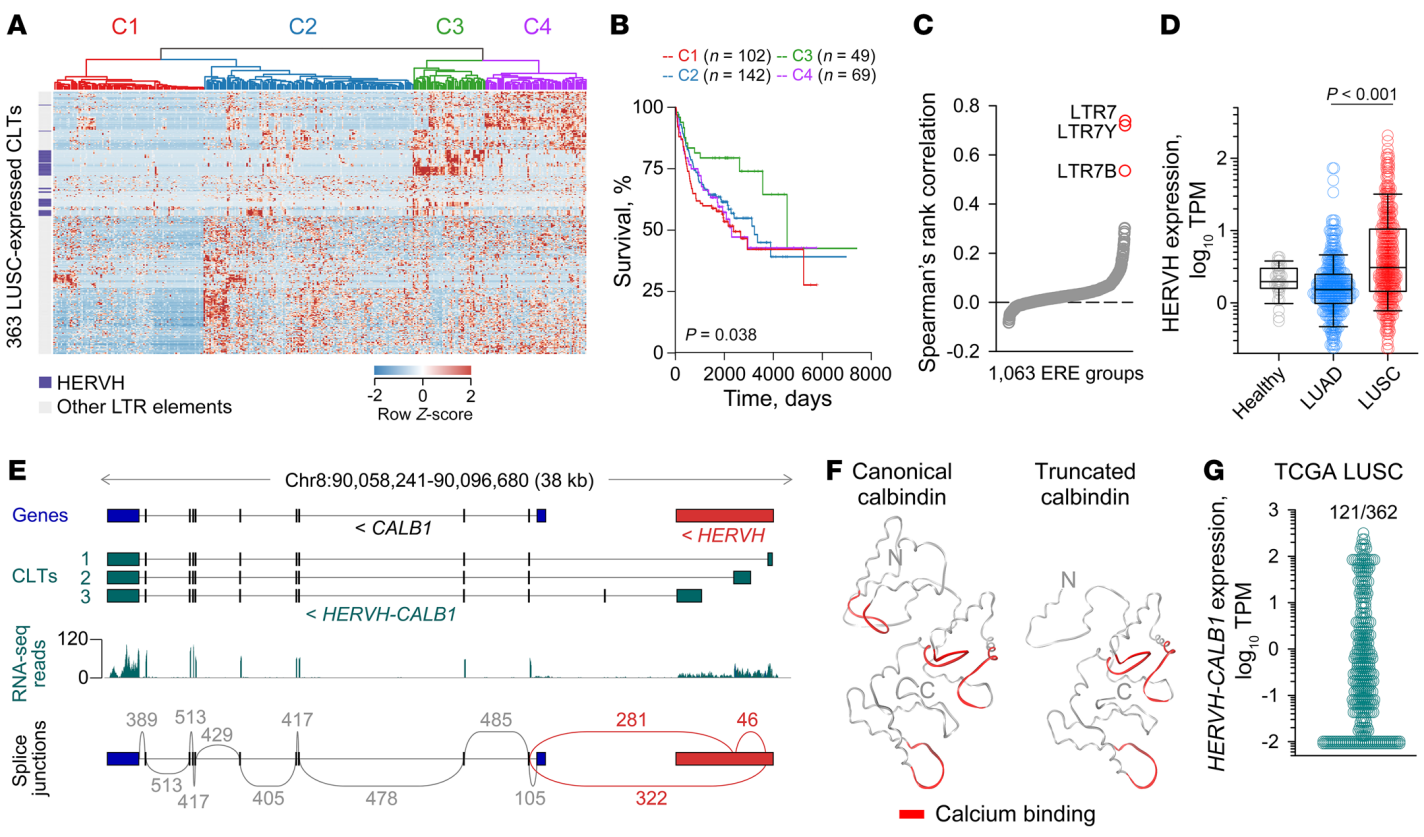
*HERVH*-driven ectopic expression of *CALB1* in LUSC. (**A**) Hierarchical clustering of TCGA LUSC samples (*P* = 362) according to expression of 363 de novo assembled cancer-specific LTR element-overlapping transcripts (CLTs) expressed in LUSC. *HERVH* elements are also indicated. (**B**) Overall survival of LUSC patients according to their assigned cluster from **A**. (**C**) Spearman’s rank correlation of transcription of 1,063 ERE groups in TCGA LUSC samples. (**D**) Combined expression of HERVH elements in healthy lung tissue (*P* = 36) or TCGA LUAD (*P* = 433) and LUSC samples (*P* = 370). *P* value calculated with 1-way ANOVA on ranks test. (**E**) Canonical GENCODE annotated transcript at the *CALB1* locus (Genes), the integrated *HERVH* provirus, assembled CLTs, RNA-Seq traces of 24 combined TCGA LUSC samples, and number of splice junctions (>40) at the same location, determined by TCGA LUSC RNA-Seq data analysis. (**F**) Modeled structures of the canonical and *HERVH-CALB1*–encoded calbindin isoforms, based on the solved structure of canonical calbindin (Protein Data Bank ID: 6FIE). (**G**) *HERVH-CALB1* expression in the TCGA LUSC cohort.

To ascertain functional consequences of ERE dysregulation in LUSC, we sought patterns of LTR element expression and association with overall survival in the LUSC cohort of The Cancer Genome Atlas (TCGA) (http://cancergenome.nih.gov). We previously identified 363 de novo assembled transcripts utilizing LTR elements and expressed specifically and recurrently in LUSC (25). Expression of these LTR element–overlapping transcripts stratified LUSC patients into 4 distinct clusters, one of which was characterized by better prognosis and higher expression of transcripts overlapping with *HERVH* proviruses (Figure 1, A and B). The latter were novel transcripts spanning *HERVH* proviruses on 6 genomic locations (Supplemental Table 1; supplemental material available online with this article; https://doi.org/10.1172/JCI164397DS1). Coregulated overall transcription was restricted to *HERVH* proviruses bearing LTR7, LTR7Y, and LTR7B LTRs independently of transcription of other EREs in LUSC (Figure 1C) and was not observed in healthy lung tissue or lung adenocarcinoma (LUAD) (Figure 1D), implying it was driven by a process specific to LUSC patients.

Two of the *HERVH*-overlapping transcripts were independently linked with LUSC survival (hazard ratio = 0.489; *P* = 0.048, log-rank test) and were transcribed from the same locus, spanning the *CALB1* gene, encoding calbindin and an upstream *HERVH* provirus (Figure 1E). Consistent with analysis of the *HERVH-CALB1* chimeric transcripts, overall *CALB1* expression was significantly correlated with better prognosis of LUSC (hazard ratio = 0.64; *P* = 0.0088m log-rank test) (Supplemental Figure 1). It was also correlated with better prognosis in cervical squamous cell carcinoma (CESC) and pancreatic adenocarcinoma (PAAD), but worse prognosis in ovarian serous cystadenocarcinoma (OV) and uterine corpus endometrial carcinoma (UCEC) (Supplemental Figure 1).

A chimeric transcript between this *HERVH* provirus and *CALB1* was cloned from the prostate cancer cell line PC3 over 30 years ago (26). Compared with the canonical calbindin, the predicted translation product of that transcript was longer, with the first 50 amino acids not corresponding to calbindin (26). However, inspection of the initially reported nucleotide sequence revealed a sequence error (missing 1 base in a quadruple G sequence at GRCh38 Chr8:90,095,448-90,095,451) that removes an early stop codon in this frame. Splicing between this *HERVH* provirus and *CALB1* has also been detected in human embryonic stem (ES) cells (27, 28), but the transcript structures were undefined. Three transcripts were assembled here (referred to as *HERVH-CALB1*), all initiated within the *HERVH* provirus upstream of *CALB1* and all including exons 2–11 of *CALB1* (Figure 1E). These transcripts partially overlapped with newly annotated transcripts ENST00000523716, ENST00000520613, and ENST00000514406, all 3 of which initiate within the *HERVH* provirus but lack 3′ end annotation (Supplemental Figure 2A).

Splice junction analysis in LUSC indicated expression of 2 (isoforms 1–2) of the 3 *HERVH-CALB1* transcripts at considerably higher levels than the annotated *CALB1* transcript (Figure 1E). *CALB1* transcription from its canonical promoter was restricted to healthy kidney and brain, as expected (29, 30), and related adrenocortical carcinoma (ACC) and brain lower-grade glioma (LGG) (Supplemental Figure 2B). *HERVH-CALB1-1* followed the same pattern and was additionally expressed in several other cancer types, including testicular germ cell tumors (TGCT) and CESC, whereas the other 2 *HERVH-CALB1* transcripts were expressed exclusively in cancer (Supplemental Figure 2B). A similar pattern of *HERVH-CALB1* expression was also observed in cell lines from the respective cancer types (Supplemental Figure 2C), indicating a cell-intrinsic property.

As *HERVH-CALB1* chimeric transcription has also been reported in human ES cells and preimplantation embryos (27, 28), we examined the expression of *HERVH-CALB1* during progressive stages of human embryogenesis, which are typically characterized by stage-specific activation of distinct HERV families (28, 31–33). Analysis of single-cell RNA-Seq data (34, 35) revealed low levels of *CALB1* transcription at the 4-cell embryo stage and in ES cells and higher transcription in the preimplantation blastocyst, which appeared to be restricted to the epiblast (Supplemental Figure 2D), in agreement with a recent report (28). Splice junction analysis indicated that *CALB1* transcription at these stages was driven by the upstream *HERVH* provirus, with the *HERVH-CALB1-1* transcript being the only one detected in epiblast cells (Supplemental Figure 2E).

All 3 *HERVH-CALB1* transcripts detected in LUSC encoded an identical protein, using a start codon in the third *CALB1* exon. The protein sequence differed from the canonical calbindin in missing the first 57 amino acids, including 1 of the 4 Ca^2+^ coordinating EF-hand domains and the putative RANBP9/IMPase-interacting domain (Figure 1F and Supplemental Figure 3). This protein sequence matched UniProt annotated protein P05937-2, which is, however, assigned to a different annotated transcript, ENST00000518457 (Supplemental Figure 2A), that was not assembled here, initiated within the second *CALB1* intron and also using the start codon in the third *CALB1* exon (36). Given that they encoded an identical protein, expression of the 3 assembled *HERVH-CALB1* transcripts was combined for subsequent analyses, which revealed high *HERVH-CALB1* expression (1–320 transcripts per million [TPM]) in 32% of the extended TCGA LUSC cohort (Figure 1G).

### HERVH-CALB1 expression promotes cancer cell–intrinsic growth.

**Figure 2:**
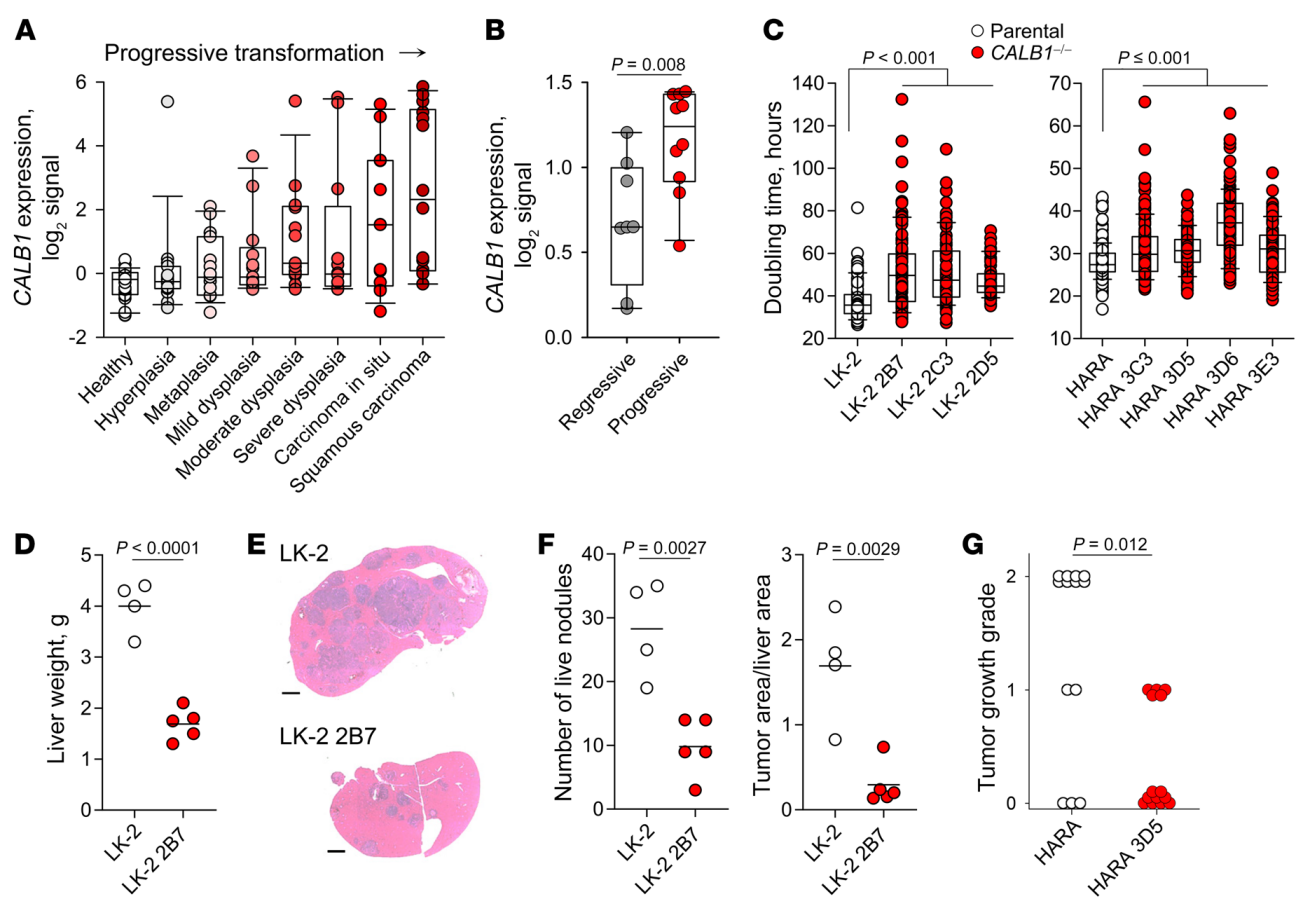
*HERVH-CALB1* expression promotes cancer cell–intrinsic growth. (**A**) *CALB1* expression in microarray data (GSE33479) from healthy lung tissue and from the indicated progressive stages preceding LUSC development. (**B**) *CALB1* expression in microarray data (GSE108082) from precancerous lesions that progressed to LUSC (progressive) or spontaneously regressed (regressive). *P* value calculated with Student’s *t* test. (**C**) In vitro doubling times of parental cells and individual calbindin-deficient clones for LK-2 (left) and HARA cells (right). *P* values calculated with 1-way ANOVA. (**D**) Liver weights of *Rag2^–/–^Il2rg^–/–^Cd47^–/–^* recipient mice injected with LK-2 or LK-2 2B7 cells. Symbols represent individual recipient mice from single experiment. *P* value calculated with Student’s *t* test. This experiment was repeated 4 times with similar results. (**E**) H&E staining of liver sections from representative mice in **D**. Scale bar: 2 mm. (**F**) Number (left) and overall size (right) of liver nodules in liver section from mice in **D**. *P* values calculated with Student’s *t* test. (**G**) Tumor growth grades in *Rag2^–/–^Il2rg^–/–^Cd47^–/–^* recipient mice injected with HARA or HARA 3D5 cells. 0, no tumors detected; 1, tumor growth only in the lung or small liver tumors; 2, extensive tumor growth in lung and liver. Symbols represent individual recipient mice pooled from 3 experiments. *P* value calculated with χ^2^ test with Yate’s correction. Uncorrected, *P* = 0.0021.

To probe a possible role for ectopic *HERVH-CALB1* expression in LUSC initiation and progression, we first examined the kinetics of its induction. In the progressive stages preceding LUSC development (37), *CALB1* expression was induced as early as metaplasia, gradually increasing to become clearly bimodal in LUSC (Figure 2A). Moreover, higher CALB1 expression was observed in precancerous lesions that progressed to LUSC than in those that regressed spontaneously (38) (Figure 2B). These 2 observations linked ectopic *CALB1* induction with more aggressive disease.

To examine the direction of causality, we performed loss-of-function experiments. Consistent with LUSC biopsies, LUSC cell lines varied in *HERVH-CALB1* expression (Supplemental Figure 4A). Exonization of the *HERVH* provirus was confirmed by reverse transcriptase–based quantitative PCR (RT-qPCR) quantitation of the *HERVH-CALB1* transcript in LK-2 and HARA cells, which expressed it at high levels (Supplemental Figure 4B). Moreover, Cas9-mediated mutation of the *HERVH* provirus demonstrated its essential promoter activity, as it diminished overall *CALB1* expression in LK-2 cells (Supplemental Figure 4C).

We further generated calbindin-deficient LK-2 and HARA cells through Cas9-mediated mutation of the *CALB1* gene to preclude use of the canonical or alternative promoters and confirmed the loss of the *HERVH-CALB1* protein product (Supplemental Figure 5). *CALB1* loss significantly delayed the in vitro doubling time of LK-2 cells as well as of the faster-growing HARA cells (Figure 2C). Moreover, the number of invasive bodies grown in 3D collagen matrices was significantly reduced by *CALB1* loss in LK-2 and HARA cells (Supplemental Figure 6). To determine whether the requirement for *CALB1* expression for maximal growth extended in vivo, we xenotransplanted LK-2 and HARA cells into immunodeficient *Rag2^–/–^Il2rg^–/–^Cd47^–/–^* recipient mice. Following intravenous injection, LK-2 cells seeded almost exclusively recipient livers and formed tumors, which were significantly smaller and less numerous for calbindin-deficient LK-2 2B7 cells (Figure 2, D–F). Intravenously injected HARA cells seeded both the lungs and livers and formed tumors in the majority, but not all, of the recipient mice (Figure 2G). Loss of *CALB1* expression in HARA 3D5 cells significantly reduced tumor burden in the livers, but not in the lungs of recipients that did develop tumors (Supplemental Figure 7). Moreover, significantly fewer mice developed tumors either in the lungs or livers when injected with HARA 3D5 than with parental cells (Figure 2G). Thus, *HERVH-CALB1* transcriptional activation in LUSC induced cancer cell–intrinsic, growth-promoting calbindin activity in vitro and in vivo despite its association with better overall survival of LUSC.

### HERVH-CALB1 activity marks squamous cell differentiation.

**Figure 3:**
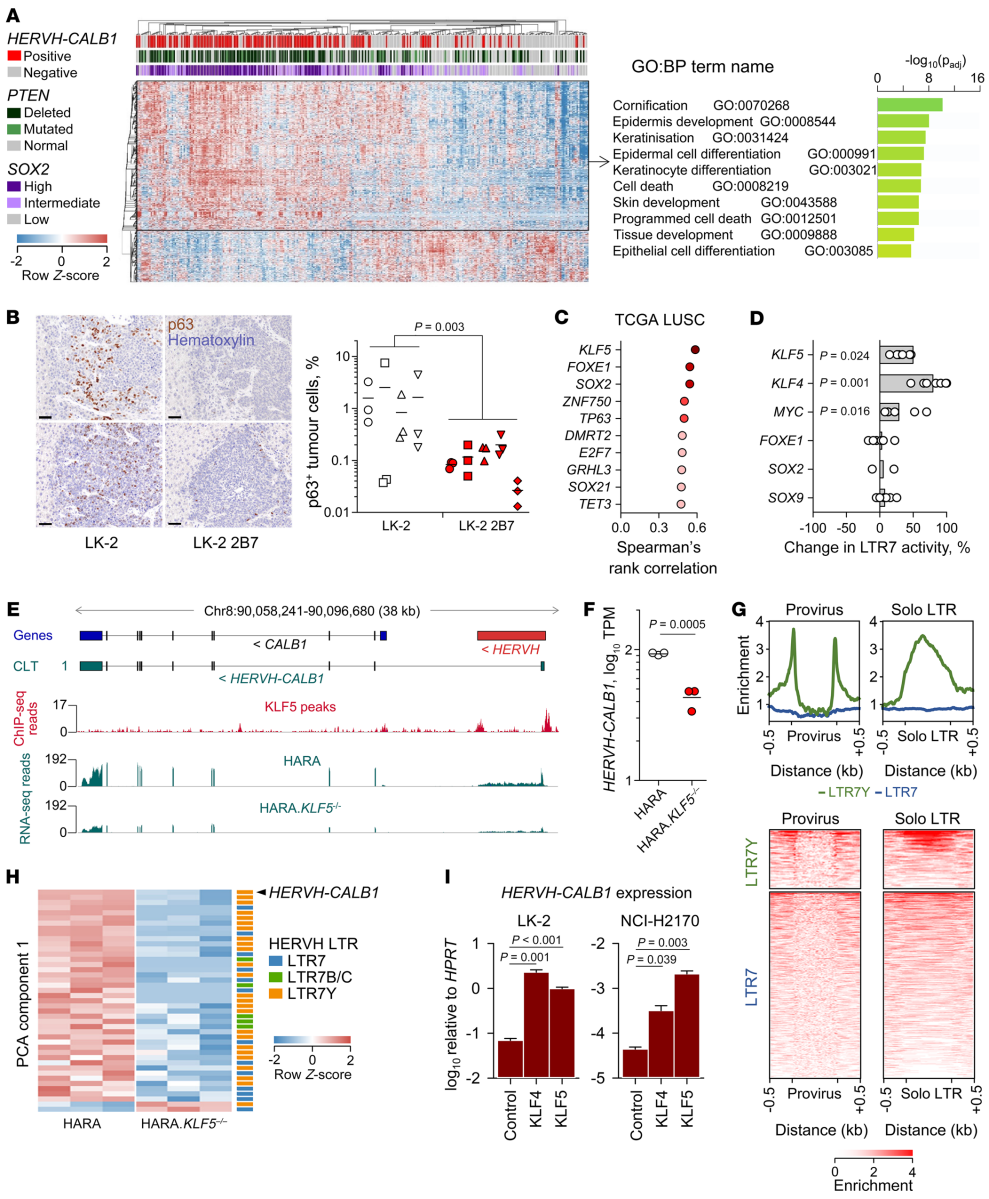
KLF5-regulated *HERVH-CALB1* activity marks squamous cell differentiation. (**A**) Hierarchical clustering of *HERVH-CALB1*–positive and –negative TCGA LUSC samples (*P* = 362) according to differential expression (≥2-fold, *q* < 0.05) of 1,526 genes (left). *PTEN* mutation status and *SOX2* expression are also indicated. Functional annotation by gene ontology (GO) of the 1,133 genes (boxed) upregulated in *HERVH-CALB1*–positive samples (right). *P* values calculated with the g:SCS algorithm. (**B**) Hematoxylin and p63 immunostaining of liver sections from *Rag2^–/–^Il2rg^–/–^Cd47^–/–^* recipients of LK-2 or LK-2 2B7 cells. Left, 2 representative mice from each group. Scale bars: 50 μm. Percentage of p63^+^ cells in LK-2 and LK-2 2B7 tumors in the same mice (right). Symbols represent individual mice with 3 regions per mouse. *P* value calculated with Mann-Whitney rank-sum test. (**C**) Spearman’s rank correlation of *HERVH-CALB1* and transcription factor expression in TCGA LUSC RNA-Seq data. (**D**) Percentage change in *HERVH* LTR7-GFP reporter activity in transcription factor–transfected HEK293T.LTR7-GFP cells, compared with untransfected HEK293T.LTR7-GFP cells. Symbols represent separate transfections. *P* values calculated with paired Student’s *t* test. (**E**) Annotated *CALB1* gene and *HERVH* provirus, *HERVH-CALB1* transcript, KFL5 peaks in ChIP-Seq data from HARA cells (GSE147853), and RNA-Seq traces of HARA cells and KFL5-deficient HARA cells (HARA.*KLF5*^–/–^) (GSE147855). (**F**) *HERVH-CALB1* expression in HARA and HARA.*KLF5*^–/–^ cells in **E**. Symbols represent experimental replicates. *P* value calculated with Student’s *t* test. (**G**) Enrichment in KLF5 binding to LTR7Y and LTR7 *HERVH* LTRs, present in full-length proviruses or as solitary (solo) LTRs, in HARA cell ChIP-Seq data (GSE147853). (**H**) *HERVH* proviruses differentially expressed between HARA and HARA.*KLF5*^–/–^ cells (GSE147855), ranked by PCA component 1 and annotated according to LTR type. (**I**) Expression of *HERVH-CALB1* determined by RT-qPCR in KLF4- or KLF5-transfected LK-2 and NCI-H2170 cells, compared with untransfected respective cells. Error bars represent the variation of 3 independent repeats. *P* values were calculated with Student’s *t* tests.

The protumor activity of calbindin expression in LUSC mirrored the action of *SOX2*, which has been established as a lineage-survival oncogene in LUSC, yet its high expression predicts better overall survival in LUSC (39–41). We therefore determined whether *HERVH-CALB1* expression was associated with defined features of LUSC. Compared with nonexpressing tumors in the TCGA LUSC data set, *HERVH-CALB1*–expressing tumors were enriched in high *SOX2* expression and in *PTEN* loss or mutation and exhibited a distinct transcriptional profile, with significant overexpression (≥2-fold; *q* < 0.05) of genes involved in cornification, keratinization, and epidermal cell differentiation (Figure 3A). This phenotype was recapitulated in LK-2 tumors that developed in *Rag2^–/–^Il2rg^–/–^Cd47^–/–^* recipient mice, where a significantly higher proportion of parental LK-2 than *CALB1*-deficient LK-2 2B7 tumor cells stained positive for p63, a marker for squamous epithelial differentiation encoded by *TP63* (Figure 3B).

In LUSC biopsies, high *HERVH-CALB1* expression was significantly correlated with expression of *KLF5*, frequently altered in LUSC (42), and of transcription factors involved in squamous epithelial differentiation, including *SOX2*, *TP63*, *GRHL3*, *ZNF750* (a TP63 target; ref. 43), and *SOX21* (a SOX2 target, ref. 44) (Figure 3C and Supplemental Figure 8). Whereas the association with *HERVH-CALB1* expression extended to other cancer types for some transcription factors (*SOX2*, *SOX21*), for others (*TP63*, *FOXE1*), it was specific to LUSC (Supplemental Figure 8). Conversely, pluripotency transcription factors, including *NANOG*, *POU5F1* (encoding OCT4), and *TFCP2L1* (encoding LBP9), that control *HERVH* activity in human ES cells (27) associated with *HERVH-CALB1* expression in other cancer types, but not in LUSC (Supplemental Figure 8). Together, these results linked *HERVH-CALB1* transcription with the transcriptional network of squamous epithelial differentiation.

To determine whether *HERVH-CALB1* transcription was directly controlled by the transcription factors orchestrating squamous epithelial differentiation, we first used a *HERVH* LTR7-GFP reporter (27), which we introduced into HEK293T cells using the Sleeping Beauty transposon system (Supplemental Figure 9A). Consistent with expression correlation (Figure 3C and Supplemental Figure 8), overexpression of KLF5 significantly increased *HERVH* LTR7 promoter activity in transduced HEK293T cells (Figure 3D). Similar results were obtained with overexpression of KLF4, a KLF5 homologue that is not typically expressed in LUSC (Figure 3D). Overexpression of MYC also increased LTR7 promoter activity, whereas overexpression of SOX2, FOXE1, or SOX9 did not (Figure 3D), in agreement with findings reported in human ES cells (27). Moreover, stable overexpression of SOX2 in HEK293T cells appeared to decrease rather than increase LTR7-GFP reporter activity (Supplemental Figure 9B). Finally, SOX2 knockdown in colorectal cancer SW620 cells, which withstand SOX2 loss (45), caused significant increase in endogenous *HERVH-CALB1* expression (Supplemental Figure 9C). These experiments suggested that SOX2 does not promote and may even inhibit *HERVH-CALB1* expression. Instead, the use of the LTR7-GFP reporter suggested that KLF5 may also control *HERVH-CALB1* expression in LUSC, particularly since the LTR7Y of the *CALB1*-associated *HERVH* provirus may be more responsive to KLF5 than the LTR7 of the LTR7-GFP reporter, as suggested by findings in human ES cells (28, 32, 33).

To obtain direct evidence for *HERVH-CALB1* control by KLF5, we analyzed KLF5 ChIP-Seq data from HARA cells (46), which revealed KLF5 binding specifically at the LTR regions of the *HERVH* provirus in the *HERVH-CALB1* locus (Figure 3E). Moreover, Cas9-mediated deletion of *KLF5* in HARA cells (46) significantly reduced expression of *HERVH-CALB1*, assessed in RNA-Seq data from the same cells (Figure 3, E and F), demonstrating the contribution of KLF5 to the maintenance of *HERVH-CALB1* transcription.

While the effect of KLF5 overexpression on LTR7-GFP reporter activity in HEK293T cells (Figure 3D) was consistent with the effect of KLF5 deletion on *HERVH-CALB1* expression in HARA cells (Figure 3, E and F), it was possible that the LTR7-GFP reporter was not faithfully capturing the full effect of KLF5 on *HERVH-CALB1* transcription. Inspection of LTR consensus sequences revealed the presence of a single perfect KLF5-binding site (with preferred bases in all positions) and 1 imperfect site (with the second preferred base in 1 position of the KLF5-binding motif) in the LTR7 consensus and the LTR7-GFP reporter construct (Supplemental Figure 10). In contrast, LTR7B, LTR7C, and LTR7Y consensus sequences contained 4, 3, and 6 perfect KLF5-binding sites, respectively (Supplemental Figure 10), suggesting that they would be more responsive to KLF5. However, the sequences of the *CALB1*-associated *HERVH* LTRs differed from the LTR7Y consensus and from each other, with the 5′ and 3′ LTRs bearing only 1 and 2 perfect KLF5-binding sites, respectively, and 2 imperfect sites each (Supplemental Figure 10), placing them between the consensus LTR7 and LTR7Y sequences.

In agreement with these predictions, in HARA cells, KLF5 bound far more strongly to LTR7Y than LTR7 *HERVH* LTRs (both in full-length proviruses and solitary LTRs) (Figure 3G). Nevertheless, several copies of the more numerous LTR7 *HERVH* proviruses were also bound by KLF5 (Figure 3G). Moreover, together with *HERVH-CALB1*, deletion of *KLF5* in HARA cells significantly reduced expression of several other LTR7Y, LTR7, LTR7B, and LTR7C *HERVH* proviruses (Figure 3H).

Given the divergence of *CALB1*-associated *HERVH* LTRs from the LTR7Y consensus and the LTR7-GFP reporter sequences, we next examined directly the effect of KLF5 overexpression on *HERVH-CALB1* expression in LUSC cell lines. KLF4 and KLF5 overexpression in LK-2 cells caused a 33-fold and 14-fold further increase, respectively, in *HERVH-CALB1* transcription (Figure 3I). Similarly, KLF4 and KLF5 overexpression in NCI-H2170 cells, which transcribe *HERVH-CALB1* minimally at steady state (Supplemental Figure 4A), induced 7-fold and 47-fold increases, respectively, in *HERVH-CALB1* transcription (Figure 3I), demonstrating that the *CALB1*-associated *HERVH* LTRs are highly responsive to KLF5.

Together, these data indicated that KLF5 displays different affinity for distinct *HERVH* LTR types, with LTR7Y being the most preferred, in agreement with studies in human ES cells (28, 32, 33). Nonetheless, they also show that KLF5 control is not restricted to LTR7Y *HERVH* proviruses and extends to *HERVH* proviruses with other LTR types. Accordingly, the LTR7-GFP reporter broadly reports KLF5 activity on HERVH LTRs as a whole as well as on *CALB1*-associated *HERVH* LTRs, which differ from the consensus LTR7Y sequence and have been reannotated as LTR7u2 (32), but likely underestimates the full effect of KLF5, particularly on conserved LTR7Y sequences.

### Cellular heterogeneity in HERVH-CALB1 expression.

**Figure 4:**
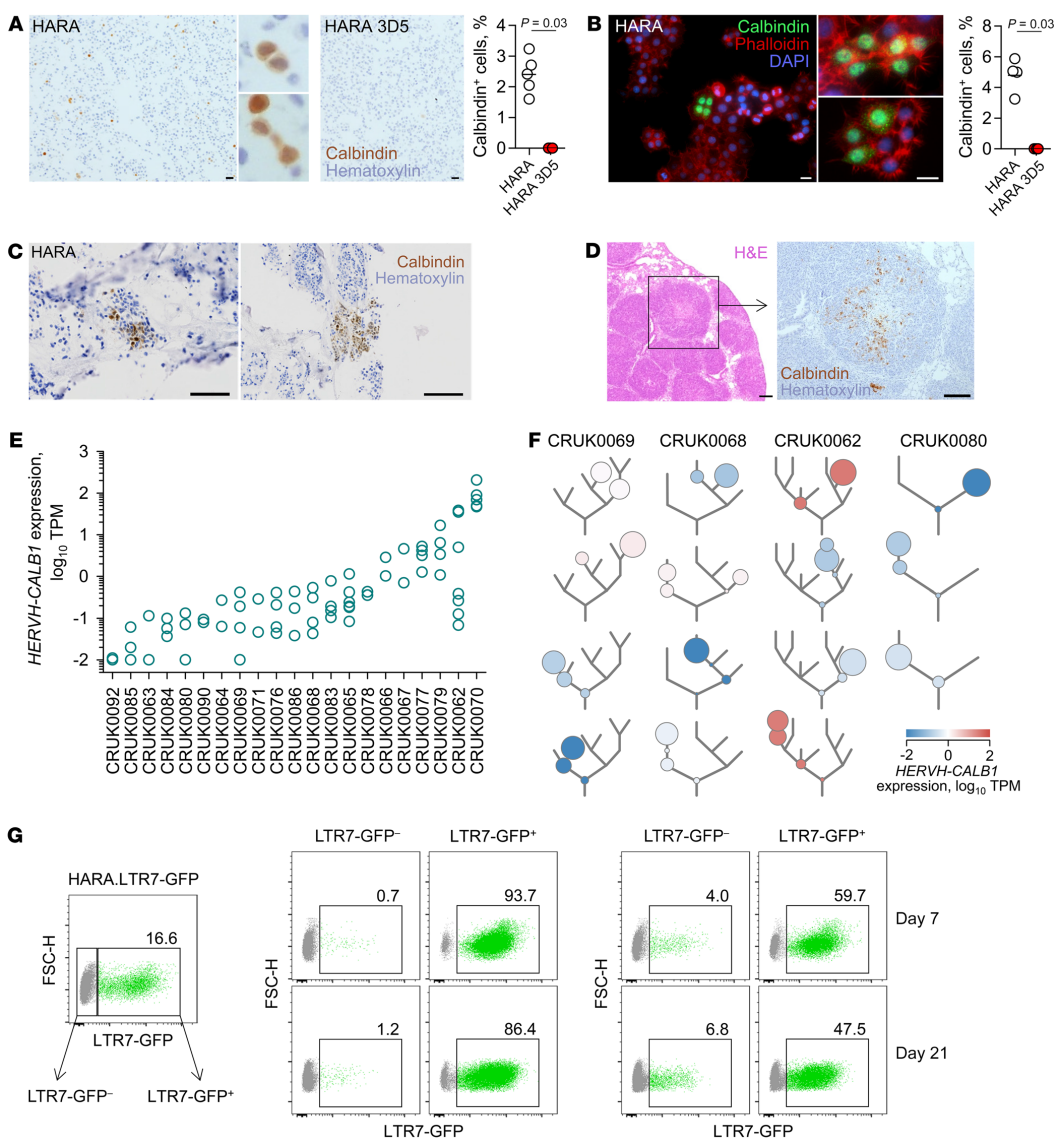
Cellular heterogeneity in *HERVH-CALB1* expression. (**A**) Immunocytochemical detection of calbindin in dissociated HARA or HARA 3D5 cell pellets (left). Scale bars: 100 μm. Insets are 10× magnified images of HARA cells. Percentage of calbindin^+^ cells in the same preparations (right). (**B**) Immunofluorescence detection of calbindin in HARA cell cultures (left). Scale bars: 20 μm. Percentage of calbindin^+^ cells in the same cultures (right). (**C**) Immunocytochemical detection of calbindin in HARA cells grown in 3D collagen matrices (left). Scale bars: 50 μm. Percentage of calbindin^+^ cells in the same cultures (right). In **A**–**C**, symbols are averages of independently acquired images. (**D**) H&E staining (left) and calbindin immunostaining (right) of sections of HARA cell tumors growing in the lungs of *Rag2^–/–^Il2rg^–/–^Cd47^–/–^* recipients. Scale bars: 200 μm. (**E**) *HERVH-CALB1* expression in TRACERx-100 LUSC patient samples (EGAD00001004591). Symbols represent individual tumor regions from each patient. Only patients with at least 2 regions sampled are shown. (**F**) *HERVH-CALB1* expression according to the evolutionary history of each region from representative samples from **E**. Circles denote the positions of the cancer cell subpopulations sampled in each region from a given patient on the constructed phylogenetic tree (gray lines) for all regions in that patient. The areas enclosed by the circles represent the proportions of each cancer cell subpopulation in the sampled region. (**G**). Flow cytometric example of gating of HARA.LTR7-GFP cells, according to GFP expression, used from the purification of positive and negative subpopulations (left). Time course of GFP expression in cells that were initially LTR7-GFP^–^ and LTR7-GFP^+^ HARA.LTR7-GFP cells in 2 separate cultures (middle and right, respectively).

The differential responsiveness of *HERVH* LTR7 to KLF5 and SOX2, despite comparable correlation of their activity with *HERVH-CALB1* expression at the biopsy level, pointed to cellular heterogeneity as one possible explanation. Immunocytochemical staining of calbindin in HARA cell pellets identified only a small fraction of strongly positive cells (2.4%), whereas calbindin-deficient HARA 3D5 cells were homogenously negative (Figure 4A). Of note, both nuclear and cytoplasmic calbindin staining were observed in HARA cells and, despite cell dissociation during cell pellet preparation, the positive cells often appeared in clusters, indicative of incomplete mitosis (Figure 4A). Similar results were obtained with immunofluorescence staining for calbindin in cultures of HARA cells, which also identified a small proportion of closely clustered positive cells (4.8%), with nuclear and cytoplasmic staining (Figure 4B). Furthermore, HARA cells grown in 3D collagen matrices also exhibited spatial heterogeneity in calbindin expression, with distinguishable clusters of positive and negative cells (Figure 4C). Consistent with nongenetic cellular heterogeneity in vitro, HARA cell tumors formed in the lungs of *Rag2^–/–^Il2rg^–/–^Cd47^–/–^* recipient mice were also heterogeneous with respect to calbindin protein, with clusters of positive and negative cells in the same tumor nodule (Figure 4D).

To measure calbindin expression heterogeneity in LUSC tumors, we examined *HERVH-CALB1* transcription in individual tumor regions sampled from LUSC patients in the TRAcking Non-small Cell Lung Cancer Evolution Through Therapy [Rx]) (TRACERx-100) cohort (47). In agreement with the TCGA LUSC cohort, 9 of the 25 (36%) LUSC patients from the TRACERx-100 cohort expressed *HERVH-CALB1* at greater than 1 TPM in 1 or more tumor regions (Figure 4E). In several tumors, individual regions differed in *HERVH-CALB1* expression by more than 1 log, although expression was rarely bimodal (Figure 4E). Moreover, *HERVH-CALB1* expression in each region tracked with the relative proportion of cancer cell subclones belonging to distinct branches of the reconstructed phylogenetic trees (Figure 4F). Nevertheless, marked differences in *HERVH-CALB1* expression were observed also between phylogenetically closely related branches (e.g., patient CRUK0062) (Figure 4F). Therefore, in vitro heterogeneity in *HERVH-CALB1* expression was also reflected in vivo.

The close proximity of calbindin-positive cells in all settings examined implied heritable *HERVH-CALB1* expression in progeny of expressing cells. Nevertheless, more dynamic fluctuation of *HERVH-CALB1* expression was also theoretically possible over longer periods. To investigate potential dynamics and stability of *HERVH-CALB1* expression over time, we introduced the *HERVH* LTR7-GFP reporter into HARA cells (Supplemental Figure 11, A and B) and monitored its expression at the single-cell level. Compared with their LTR7-GFP^–^ counterparts, LTR7-GFP^+^ HARA cells were enriched for *HERVH-CALB1* expression, assessed by the levels of calbindin (Supplemental Figure 11C), indicating a degree of faithfulness of the reporter. Following a 4-week culture of FACS-purified LTR7-GFP^+^ HARA cells, only a fraction (15%) of previously GFP^+^ cells retained GFP expression (Figure 4G). A similar pattern was observed following subsequent rounds of selection for LTR7-GFP^+^ cells, which begun to lose GFP expression (Figure 4G). In contrast, a fraction of FACS-purified LTR7-GFP^–^ HARA cells from the same population of previously GFP^+^ cells begun to reexpress GFP (Figure 4G), consistent with dynamic regulation of *HERVH* LTR7-GFP expression. Together, these results indicate that *HERVH* LTR7 activity and, consequently, *HERVH-CALB1* expression fluctuated over time in individual HARA cells, but once induced, it remained stable over multiple cell divisions.

**Figure 5:**
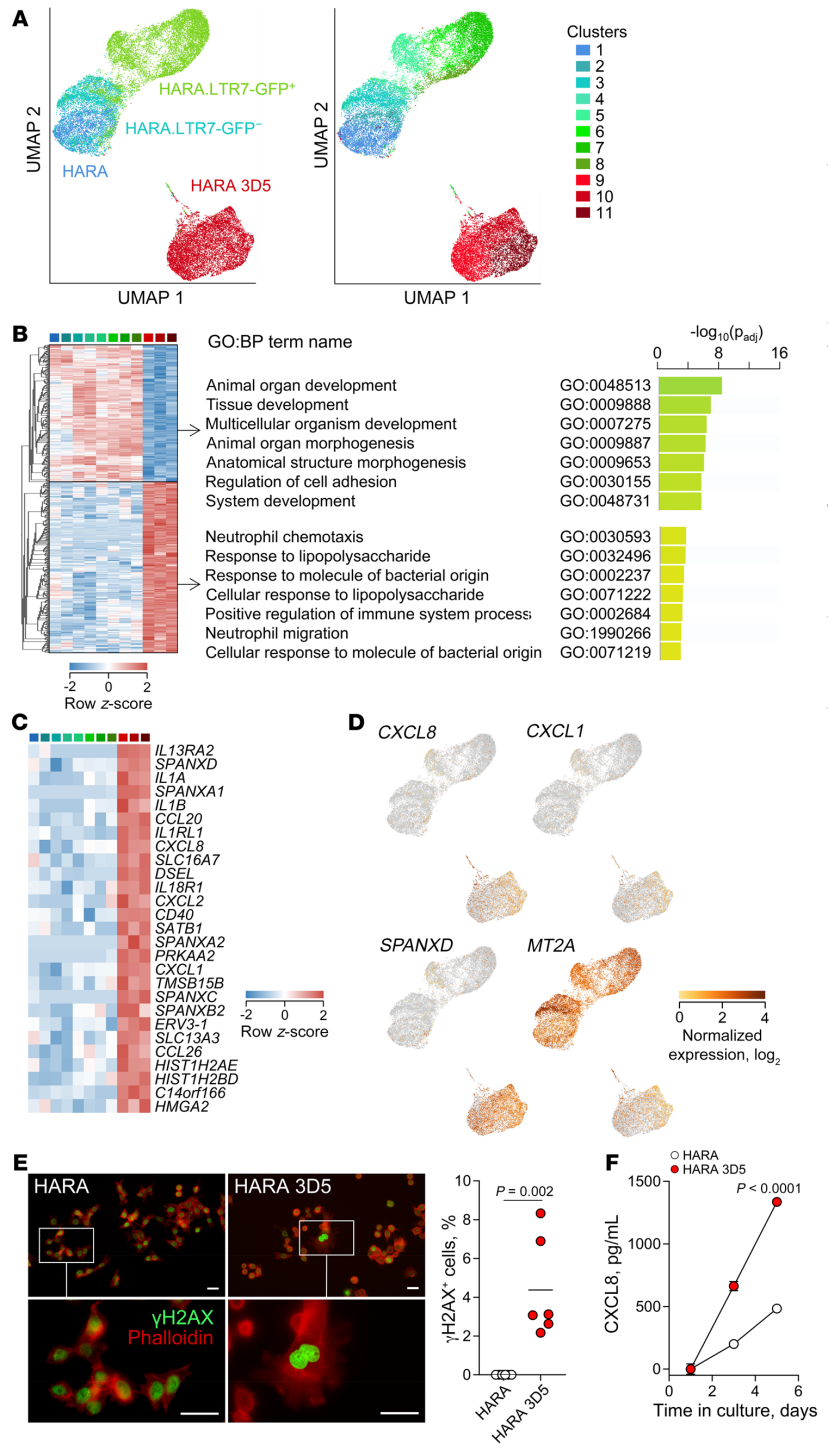
Lack of *HERVH-CALB1* expression associates with cellular senescence. (**A**) UMAP clustering of HARA cells, calbindin-deficient HARA 3D5 cells, HARA.LTR7-GFP^+^ cells, and HARA.LTR7-GFP^–^ cells according to scRNA-Seq profiling, labeled by their genotype/phenotype (left) or by their assigned cluster (right). (**B**) Heatmap of expression of 566 genes differentially expressed (≥2-fold, *q* < 0.05) between HARA 3D5 cell clusters (clusters 9–11) and all other cell clusters from **A** (left), and functional annotation by GO of the upregulated and downregulated genes in this comparison (right). *P* values calculated with the g:SCS algorithm. (**C**) Heatmap of expression of selected genes upregulated in HARA 3D5 cell clusters, ordered by fold change in expression in the cell clusters from **A**. (**D**) Normalized expression of *CXCL8*, *CXCL1*, *SPANXD*, and *MT2A* in UMAP cell cluster projections as in **A**. (**E**) γH2AX and phalloidin staining of HARA and HARA 3D5 cells (left). Lower panels show magnified images of the indicated regions in the upper panels. Scale bars: 20 μm. Percentage of γH2AX ^+^ cells, additionally exhibiting signs of DNA damage, in the same preparations (right). Symbols represent individual regions of interest. (**F**) Mean CXCL8 concentration (±SEM) over time in the supernatants of HARA and HARA 3D5 cells, determined by ELISA (*P* = 6 per time point). *P* value calculated with Student’s *t* test. One representative of 3 independent experiments is shown.

To explore cellular phenotypes associated with *HERVH-CALB1* expression, we analyzed the transcriptional profile of HARA cells according to *HERVH-CALB1* or *HERVH* LTR7-GFP reporter expression at the single-cell level. To this end, parental HARA cells were compared with calbindin-deficient HARA 3D5 cells as well as LTR7-GFP^+^ and LTR7-GFP^–^ HARA cells by single-cell RNA-Seq (Figure 5A). Transcription of *HERVH-CALB1* was detected in approximately 30% of HARA cells, and in LTR7-GFP^+^ HARA cells, it was correlated with *LTR7-GFP* transcription (Supplemental Figure 12, A–C). Substantially higher *HERVH-CALB1* mRNA positivity, despite the high drop-out rate associated with single-cell RNA-Seq, than calbindin protein positivity (Figure 4, A–C) may reflect relative insensitivity of calbindin staining (particularly of the truncated protein isoform) or variable levels of mRNA expression and subsequent protein expression in the population as a result of the epigenetic state of the *HERVH* provirus driving *CALB1* expression or availability of necessary transcription factors. Although the role of chromatin accessibility in the heterogeneity of *HERVH-CALB1* expression could not be directly addressed at this point, owing to lack of single-cell epigenetic data, transcriptional comparison of *CALB1* mRNA-positive (*CALB1*^+^) and *CALB1* mRNA-negative (*CALB1*^–^) HARA cells indicated differences in transcription factor expression (Supplemental Figure 12D). Indeed, compared with their *CALB1*^–^ counterparts, *CALB1*^+^ LTR7-GFP^+^ HARA cells expressed higher levels of *LTR7-GFP* reporter transcripts, further supporting coregulation of the two, as well as of *KLF5* and *MYC* (Supplemental Figure 12D). Similar results were also obtained with comparison of *CALB1*^+^ and *CALB1*^–^ HARA 3D5 cells (Supplemental Figure 12D), in which Cas9-mediated mutation of the *CALB1* gene precludes downstream effects of calbindin expression. In HARA 3D5 cells, lack of *CALB1* expression was also associated with reduced expression of *SPRR2A*, *SPRR2D*, and *KRT16* (Supplemental Figure 12E), which have been previously identified as part of KLF5-dependent murine epithelial differentiation programs (48, 49). These results suggest that cellular heterogeneity in *HERVH-CALB1* expression could arise from variable KLF5 activity, which could operate in concert with chromatin accessibility states, particularly since KLF5 has been recently shown to render chromatin at LTR7Y *HERVH* loci more accessible to other transcription factors in human ES cells (33).

### HERVH-CALB1 expression protects from cellular senescence.

At the single-cell transcriptome level, individual HARA cells distinctly segregated according to genotype and LTR7-GFP reporter activity, with subclusters identified within each population (Figure 5, A and B). However, calbindin-deficient HARA 3D5 cells were substantially and equally divergent from all the calbindin-sufficient HARA cell subclusters irrespective of *HERVH-CALB1* expression in the latter (Figure 5, A and B). This finding suggested that calbindin expression in a proportion of HARA cells at any one time conferred a transcriptional profile to the whole population that clearly distinguished them from a population of HARA 3D5 cells unable to express calbindin. Differences belonged to 2 major pathways depending on the direction of transcriptional change. Gene transcripts upregulated in calbindin-sufficient cells were primarily involved in developmental processes (Figure 5B), in line with a role for calbindin in squamous epithelial differentiation (Figure 3, A–C). In contrast, gene transcripts upregulated in calbindin-deficient cells were linked with innate immune responses to microbial products and neutrophil chemotaxis (Figure 5B). Inspection of the latter set of genes revealed significant upregulation of several neutrophil chemoattractants, including *CXCL8*, *CXCL1*, and *CXCL2* and other immune mediators and receptors, specifically in calbindin-deficient HARA 3D5 cells (Figure 5, C and D). Although these immune mediators are produced in response to foreign microbes, a stimulus that was not present in our system, they are also part of SASP, a proinflammatory phenotype that accompanies cellular senescence (3, 50). Indeed, the chemokine CXCL8 (also known as IL-8) is considered a prototypic marker and main mediator of SASP (3, 50), which is accompanied by elevated expression of additional immune mediators, including the upregulated chemokines and cytokines *CXCL1*, *CXCL2*, *IL1A*, *IL1B*, *CCL20*, and *CCL26* and the cytokine receptor *IL13RA2* (51).

Also noticeable were gene transcripts involved in nuclear organization and integrity. These included *SPANXD* and other SPANX (sperm protein associated with the nucleus on the X chromosome) family members (Figure 5, C and D), whose expression is restricted to spermatozoa and cancer cells (52). Through interaction with lamin A, SPANX proteins have recently been shown to preserve nuclear architecture and prevent senescence and SASP in melanoma cell lines (53). They also included *SATB1*, encoding a nuclear matrix protein whose expression correlates with life span in mice (54) and prevents cellular senescence in neurons (55) as well as *HIST1H2AE*, *HIST1H2BD*, and *HMGA2* (Figure 5, C and D), encoding histones and the nonhistone chromosomal high-mobility group protein, respectively. Overexpression of such nuclear integrity components likely represented a compensatory response to senescence calbindin-deficient HARA 3D5 cells, which was, however, insufficient to fully prevent growth defects or SASP. Also likely compensatory was the upregulated transcription in calbindin-deficient HARA 3D5 cells of genes encoding calcium-regulated proteins, including *CALM1*, encoding calmodulin 1, *S100A2*, and *CALR*, encoding calreticulin (Supplemental Figure 12F). Notably, such calcium-regulated proteins are considered instrumental in the induction of senescence by elevated intracellular Ca^2+^ levels (56).

In contrast, calbindin-deficient HARA 3D5 cells showed significantly reduced expression of *MT2A* (Figure 5, C and D), encoding metallothionein-2A, a cellular stress–induced antiinflammatory antioxidant and one of very few proteins whose overexpression counteracts senescence and extends life span across phyla (57). Collectively, the data indicate that loss of calbindin activity in HARA 3D5 cells induces cellular senescence, with accompanying compensatory phenotypes and SASP.

Consistent with the results of transcriptional profiling, *CALB1* deficiency reduced the metabolic activity of HARA (Supplemental Figure 13). Moreover, calbindin-deficient HARA 3D5, but not parental HARA cells, exhibited evidence for DNA damage, as indicated by the presence of enlarged nuclei stained positive for the phosphorylated histone variant 2AX (γH2AX) (Figure 5E). Finally, we measured levels of SASP prototypic chemokine CXCL8 in the supernatants of HARA and calbindin-deficient HARA 3D5 cell cultures. Despite their slower growth compared with parental HARA cells (Figure 2C), HARA 3D5 cells secreted substantially higher levels of CXCL8, which accumulated over time in culture supernatants (Figure 5F). These findings support a role for the *HERVH-*driven, ectopically expressed calbindin isoform in protection from senescence of LUSC cancer cells in agreement with an involvement of the canonical calbindin in protection from senescence and apoptosis of nontransformed human and mouse cell types, where it is normally expressed (58, 59), and of ovarian cancer cells (60).

To assess whether the *HERVH-*driven calbindin isoform was functionally equivalent to the canonical isoform, we stably expressed each isoform in calbindin-deficient HARA 3D5. As detection of calbindin with the available polyclonal antibody underestimates the abundance specifically of the *HERVH-*driven calbindin isoform owing to its N-terminal truncation (Methods), we ensured equivalent expression of the 2 isoforms in transduced HARA 3D5 cells by flow cytometric measurement of GFP expression, driven by an internal ribosome entry site (IRES) in the transducing vectors as well as by quantifying overall *CALB1* and *IRES* transcription by RT-qPCR in the same cells (Supplemental Figure 14, A and B). Immunofluorescence staining for calbindin showed expression of each isoform in all transduced cells, with the protein restricted predominantly to the cytoplasm (Supplemental Figure 14C). This pattern contrasted with the distribution of endogenously expressed *HERVH-*driven calbindin in HARA cells, which was also found in the nucleus of recently divided cells (Figure 4, A and B), suggesting an effect of cell division. Notably, whereas the stably expressed canonical isoform exhibited diffuse cytoplasmic localization, the stably expressed *HERVH-*driven isoform showed more punctate localization (Supplemental Figure 14C), similar to the endogenously produced isoform (Figure 4B). Nevertheless, both isoforms significantly suppressed the induction of CXCL8 release caused by calbindin deficiency in HARA 3D5 cells, with the *HERVH-*driven isoform being marginally more efficient than the canonical isoform in reverting this phenotype (Supplemental Figure 14D). These findings indicate that, despite the loss of one EF-hand domain, the *HERVH-*driven calbindin isoform was at least as efficient as the full-length isoform in preventing SASP and associated CXCL8 release.

### HERVH-CALB1 expression averts protumor inflammation.

A role for calbindin in preventing senescence is consistent with its protumor effects on cancer cell–intrinsic growth and squamous epithelial differentiation, indicated by observations in vitro, in xenotransplantation, and in preinvasive lesions preceding LUSC development. However, such a role for calbindin was seemingly at odds with the association of *HERVH-CALB1* expression with better overall survival of fully developed LUSC. While cellular senescence is inherently a tumor-suppressive mechanism, SASP-driven inflammation may affect tumor growth indirectly through modulation of antitumor immunity (3). We hypothesized that this functional dichotomy could underlie the contrasting protumor and antitumor associations of *HERVH-CALB1* expression in LUSC. We therefore examined immune features associated with *HERVH-CALB1* expression.

**Figure 6:**
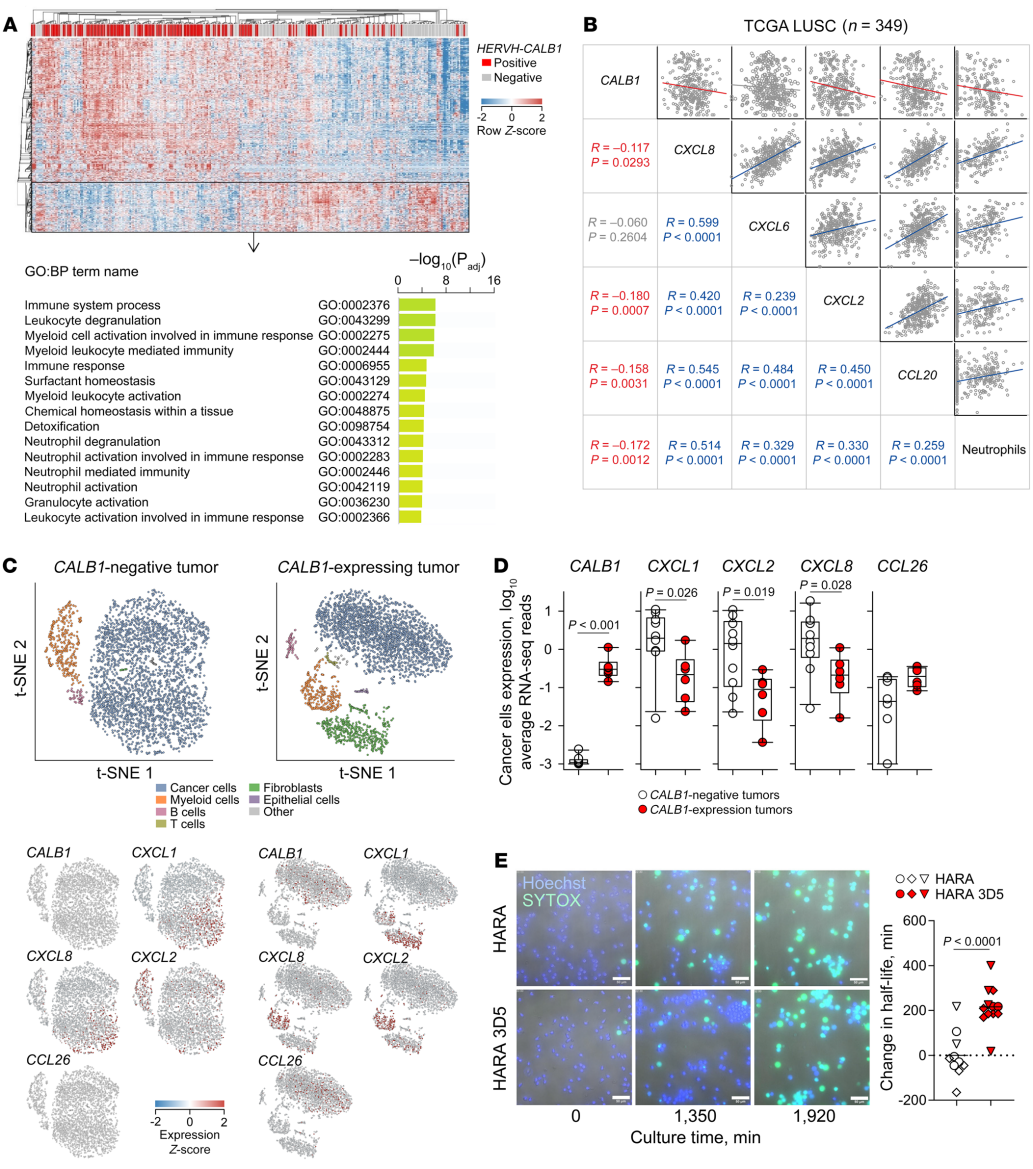
*HERVH-CALB1* expression controls cancer cell–intrinsic chemokine production. (**A**) Hierarchical clustering of *HERVH-CALB1*–positive and –negative TCGA LUSC samples (*P* = 362) according to differential expression (≥2-fold, *q* < 0.05) of 1,526 genes from Figure 3A is shown here again (top) to indicate the set of 393 genes (boxed) downregulated in *HERVH-CALB1*–positive compared with *HERVH-CALB1*–negative samples. Functional annotation by GO of the 393 genes downregulated in *HERVH-CALB1*–positive samples (bottom). *P* values calculated with the g:SCS algorithm. (**B**) Correlation between expression of *CALB1* and the indicated chemokine/cytokine or inferred neutrophil proportion in RNA-Seq from TCGA LUSC samples (*P* = 349). Blue and red colors indicate significant positive and negative correlation, respectively, and gray color indicates lack of significant correlation. (**C**) t-Distributed stochastic neighbor embedding (t-SNE) clustering of distinct cell types (top) and expression of *CALB1* or of the indicated chemokines/cytokines in projections of the same clusters (bottom) determined by analysis of scRNA-Seq data (GSE148071) from representative *CALB1*-negative and *CALB1*-positive LUSC tumors. (**D**) Cancer cell–intrinsic expression of *CALB1* and of the indicated chemokines/cytokines in *CALB1*-negative (*P* = 10) and *CALB1*-positive (*P* = 6) LUSC tumors as in **C**. Symbols represent tumors from individual patients. *P* values calculated with Student’s *t* test. (**E**) Examples of DNA staining of all cells (DAPI) and of cells with compromised plasma membranes (SYTOX) in human neutrophil cultures incubated over time with supernatants from HARA and HARA 3D5 cells (left). Scale bars: 50 μm. Change in half-life of neutrophils incubated with HARA 3D5 cell supernatants compared with those incubated with HARA cell supernatants (right). Three independent HARA and HARA 3D5 cell culture supernatants were tested, indicated by different symbols. The same symbols are used for separate fields of view of each neutrophil culture. One of 2 experiments is shown. *P* value calculated with Student’s *t* test.

Whereas *HERVH-CALB1*–expressing TCGA LUSC biopsies overexpressed genes involved in epidermal cell differentiation (Figure 3A), those lacking *HERVH-CALB1* expression were marked by significant overexpression (≥2-fold, *q* < 0.05) of genes involved in activation of neutrophils and other myeloid cells (Figure 6A). Expression levels of *CXCL8*, *CXCL2*, *CXCL6*, and *CCL20* were positively correlated with each other as well as with the fraction of neutrophils in TCGA LUSC samples (Figure 6B). In contrast, levels of *HERVH-CALB1* were negatively correlated with *CXCL8* and *CXCL2* expression and with the neutrophil fraction in the same samples (Figure 6B).

Spontaneous regression or progression of airway lesions preceding LUSC development has recently implicated immune surveillance mechanisms (61). Notably, in contrast with other proinflammatory cytokines preferentially expressed in regressive lesions, *CXCL8* was found preferentially expressed in progressive lesions, where it was strongly correlated with myeloid cell infiltration (61). Consistent with its higher expression in progressive than in regressive lesions (Figure 2B), *CALB1* expression displayed a strong positive correlation with *CXCL8* expression in these preinvasive lesions (Supplemental Figure 15). Levels of *CALB1* transcription were undetectable in healthy airways, but gradually increased in preinvasive airway lesions, according to progressive transformation to LUSC (Figure 2A). *CXCL8* transcription was already detectable in healthy airways, where it was physiologically produced, and its levels rose in proportion with *CALB1* transcription in preinvasive lesions, but this correlation was no longer positive in fully developed LUSC (Supplemental Figure 15).

CXCL8 is a potent chemoattractant for neutrophils and myeloid-derived suppressor cells and is linked with worse prognosis in multiple cancer types, including lung cancer (62, 63). However, CXCL8 and other key neutrophil chemoattractants can be produced by cancer cells as well as a variety of stroma and immune cells, notably neutrophils and other myeloid cells themselves (64), and its predominant source may change over the course of transformation (62, 63).

To determine the contribution of cancer cells and the tumor microenvironment to chemokine/cytokine production, we analyzed single-cell RNA-Seq data from LUSC biopsies (65). In biopsies lacking *CALB1* expression, cancer cells not only transcribed *CXCL1*, *CXCL2*, *CXCL8*, and other immune mediators; they were also the predominant source (Figure 6C and Supplemental Figure 16). In contrast, in *CALB1*-expressing tumors, transcription of the same mediators was minimal in cancer cells and instead was found in myeloid cells (*CXCL2*, *CXCL8*) and/or tumor-associated fibroblasts (*CXCL5*, *CXCL6*) (Figure 6C and Supplemental Figure 16). Exceptions to this pattern were *IL1B*, which was transcribed predominantly by myeloid cells irrespective of the *CALB1* status of the tumor, and *CCL26*, which was transcribed predominantly by *CALB1*-expressing cancer cells (Figure 6C and Supplemental Figure 16). Moreover, expression of *CALB1* in cancer cells exhibited a highly significant inverse correlation with cancer cell–intrinsic expression of *CXCL1*, *CXCL2*, and *CXCL8* and a positive correlation with CCL26 expression, which did not, however, reach statistical significance (*P* = 0.057) (Figure 6D). Thus, cancer cells were the main producers of *CXCL1*, *CXCL2*, and *CXCL8*, but cancer cell–intrinsic production of these chemokines was prevented by *CALB1* expression.

To further probe a direct link between cancer cell–intrinsic *HERVH-CALB1* and *CXCL8* expression, we examined RNA-Seq data from in vitro–grown lung squamous and adenosquamous cancer cell lines, where the confounding effects of tumor heterogeneity or purity can be excluded. In agreement with TCGA LUSC biopsies, expression of *CXCL8*, *CXCL2*, *CXCL6*, and *CCL20* in individual cancer cell lines was positively correlated, whereas *HERVH-CALB1* expression was negatively correlated with *CXCL8* and *CXCL2* expression (Supplemental Figure 17), consistent with control of neutrophil chemoattractant production by calbindin in a cancer cell–intrinsic manner.

Loss-of-function experiments demonstrated a role for calbindin in regulating CXCL8 secretion,as part of SASP in HARA cells. To determine whether this effect of calbindin on SASP-related chemokine expression, assessed by transcriptional profiling (Figure 5, C and D), and secreted CXCL8 levels, assessed by ELISA (Figure 5F), was sufficient to modulate neutrophil behavior, we tested the activity supernatants of HARA and calbindin-deficient HARA 3D5 cell cultures directly on primary neutrophils from healthy donors. Indeed, supernatants from calbindin-deficient HARA 3D5 cells significantly extended the half-lives of human neutrophils isolated from healthy donors when compared with supernatants from HARA cells (Figure 6E), consistent with a neutrophil-supporting activity of SASP chemokines. Collectively, these results supported a model whereby expression of calbindin in lung squamous cancer cells counteracted cellular senescence and ensuing SASP, which would otherwise promote neutrophil-dominated protumor inflammation.

## Discussion

Senescence likely evolved as a tumor-suppressive mechanism, intrinsically arresting proliferation of transformed cells and inducing paracrine senescence in neighboring cells, and its escape may therefore be a necessary step in cancer initiation (3, 50). However, an important protumor component of senescence, particularly through SAPS, is increasingly recognized (3). *HERVH*-driven ectopic *CALB1* expression is at the crossroads of LUSC evolution and appears to display antagonistic pleiotropy. Intraclonal competition and the need to escape senescence would select for *HERVH*-*CALB1* expression. However, the early cancer cell–intrinsic advantage afforded by *HERVH*-*CALB1* expression is ultimately offset by the suppression of protumor inflammation, which would otherwise compromise extrinsic tumor control.

Calbindin is thought to preserve cellular fitness and replicative capacity by buffering intracellular Ca^2+^, elevated levels of which are a trigger and hallmark of senescence (56). Our findings indicate that the *HERVH-*driven calbindin isoform retains this function of the canonical calbindin isoform, as measured by the prevention of SASP and associated CXCL8 release, despite the loss of one EF-hand domain. However, alternative Ca^2+^-sensing functions have also been proposed for calbindin, with the identification of several binding partners (66, 67). While the physiological relevance of calbindin-binding partners remains unclear (66, 67), recent in vitro studies support an oncogenic role for the canonical isoform of calbindin, with one study implicating binding to the E3 ubiquitin ligase MDM2 (60, 68, 69). Moreover, chimeric *HERVH-CALB1* transcripts detected in human ES cells and preimplantation embryos raise the possibility that ectopic expression of calbindin, particularly in the epiblast as detected here and in a recent report (28), may represent an exaptation event that contributes to normal human embryo development.

Distinct *HERVH* subfamilies exhibit stage-specific expression during human embryogenesis, owing to differential responsiveness of provirus LTRs to transcription factors, with LTR7 and LTR7Y *HERVH* provirus expression enriched in primed and naive ES cells, respectively (31, 33). Despite being driven by LTR7Y, the expression of *HERVH-CALB1* does not match precisely the pattern of the LTR7Y *HERVH* subfamily (31, 33). However, using phyloregulatory analysis, Carter et al. defined 8 distinguishable *HERVH* LTR7 subfamilies, with over half of LTR7Y members, including the *CALB1*-associated *HERVH* provirus, reannotated as LTR7u2, a subfamily expressed specifically in the pluripotent epiblast (32).

While elucidation of the precise function(s) and putative binding partners of *HERVH*-driven calbindin in other settings will require further investigation, a clear outcome of the *HERVH*-driven isoform is the control of cancer cell–autonomous CXCL8 secretion both in vitro and in vivo. In the absence of calbindin, cancer cells become the dominant source of CXCL8 as well as of other potent neutrophil chemoattractants in fully transformed tumors. In contrast, in preinvasive lesions (61) and in calbindin-expressing LUSC tumors, these chemokines are produced predominantly by myeloid cells themselves, with cancer cells expressing only the monocyte chemoattractant CCL26. Thus, a calbindin-dependent shift in the production of neutrophil chemoattractants over the course of tumor progression may determine the tumor immune contexture and, consequently, the rate of tumor progression.

In addition to neutrophil chemoattraction, CXCL8 and other CXC chemokines exhibit wider functions in cancer, directly promoting angiogenesis (62, 70) and cancer cell growth (71, 72) and, notably, reinforcing senescence in a self-amplifying manner (73). Given the growth defects of *CALB1*-deficient LUSC cell lines, autocrine CXCL8 signaling driving cancer cell–intrinsic growth is unlikely to compensate for this proliferative disadvantage or to explain the worse prognosis of *CALB1*-negative tumors. Nevertheless, paracrine CXCL8 growth signals on nonsenescent cancer cells or angiogenic signals on endothelial cells may indeed contribute to the poorer outcome of *CALB1*-negative tumors.

Additional protumor effects of CXCL8 notwithstanding, neutrophil recruitment has been strongly associated with worse prognosis in human and animal studies (62, 74–76). Although mice lack CXCL8, other CXC chemokines, such as mouse CXCL5, the presumed orthologue of human CXCL6, signaling via the common receptor CXCR2 have been demonstrated as promoting lung cancer growth through neutrophil recruitment (75, 76), and mice with restored ability to produce CXCL8 are more susceptible to carcinogenesis through myeloid cell recruitment (77).

Together, our data support a model whereby ectopic expression of a *HERVH*-driven calbindin isoform prevents cancer cell senescence and associated inflammation. In light of recent data incriminating ERE derepression as the trigger of both cancer and age-related inflammation (17, 18), *HERVH*-driven *CALB1* expression may represent a cooption of one retroelement in countering the collective action of many others.

## Methods

An expanded Methods section, including full, uncut gels, is available in the Supplemental Methods.

### Mice.

*Rag2^–/–^Il2rg^–/–^Cd47^–/–^* mice (B6.129S-*Rag2^tm1Fwa^ Cd47^tm1Fpl^ Il2rg^tm1Wjl^*/J) were originally obtained from the Jackson Laboratory (strain 025730) and were subsequently maintained at The Francis Crick Institute Biological Research Facility under specific pathogen–free conditions. Eight- to fourteen-week-old male or female mice were used for all experiments, randomly allocated to the different groups.

### Cell lines.

HEK293T (CVCL_0063) cells, squamous cell lung carcinoma HARA (CVCL_2914) and LK-2 (CVCL_1377) cells, and colorectal cancer SW620 (CVCL_0547) cells were obtained from, verified as mycoplasma free, and validated by DNA fingerprinting by the Cell Services facility at The Francis Crick Institute. HARA and LK-2 cells were originally sourced from the Japanese Collection of Research Bioresources (JCRB) Cell Bank (JCRB1080.0 and JCRB0829, respectively) and were deposited with the Cell Services Facility at The Francis Crick Institute. HARA cells were grown in RPMI 1640 Medium (Thermo Fisher Scientific) with 10% fetal bovine serum, and unless otherwise indicated, other cell lines were grown in IMDM (Sigma-Aldrich) supplemented with 5% fetal bovine serum (Thermo Fisher Scientific). Media were further supplemented with l-glutamine (2 mmol/L, Thermo Fisher Scientific), penicillin (100 U/mL, Thermo Fisher Scientific), and streptomycin (0.1 mg/mL, Thermo Fisher Scientific).

### Transcript identification and bulk RNA-Seq read mapping and quantitation.

Transcripts were previously de novo assembled on a subset of the RNA-Seq data from TCGA (25). Samples from TCGA were downloaded through the *gdc-client* application, and the .*bam* files were parsed with a custom Bash pipeline using GNU parallel (78). RNA-Seq data from TCGA, the Genotype-Tissue Expression Project (GTEx) (https://gtexportal.org), the Cancer Cell Line Encyclopedia (CCLE) (https://sites.broadinstitute.org/ccle), and TRACERx-100 (EGAD00001004591) were mapped to our de novo cancer transcriptome assembly and counted as previously described (25). Briefly, TPM values were calculated for all transcripts in the transcript assembly (25), with a custom Bash pipeline and Salmon (79), version 0.12.0, which uses a probabilistic model for assigning reads aligning to multiple transcript isoforms, based on the abundance of reads unique to each isoform (79). We separately quantified expression of annotated genes by using a transcript index with all GENCODE transcript_support_level:1 entries and collapsing counts for the same gene. Splice junctions were visualized using the Integrative Genome Viewer (IGV) (80), version 2.4.19.

### Data availability.

HARA cell subline scRNA-Seq data generated in this study were deposited at the EMBL-EBI repository (www.ebi.ac.uk/arrayexpress) (E-MTAB-11550). Previously published data are available at TCGA, GTEx, CCLE, and TRACERx-100 (EGAD00001004591) consortia and in the NCBI’s Gene Expression Omnibus database (GEO GSE33479, GSE108082, GSE147853, GSE147855, and GSE148071) for individual studies. The TCGA and GTEx data used for the analyses described in this manuscript were obtained from dbGaP accession nos. phs000178.v10.p8.c1 and phs000424.v7.p2.c1 in 2017. Values for all data points found in graphs can be found in the supporting data values file.

### Statistics.

Statistical comparisons were made using GraphPad Prism, version 9 (GraphPad Software), or SigmaPlot, version 14.0 (Systat Software). Parametric comparisons of normally distributed values that satisfied the variance criteria were made by unpaired 2-tailed Student’s *t* tests or ANOVA. Data that did not pass the variance test were compared using nonparametric 2-tailed Mann-Whitney rank-sum tests or ANOVA on ranks tests. *P* values of less than 0.05 were considered statistically significant. Statistical analyses of RNA-Seq data were carried out using Qlucore Omics Explorer, version 3.8 (Qlucore).

### Study approval.

All experiments were approved by the ethics committee of the Francis Crick Institute and conducted according to local guidelines and UK Home Office regulations under the Animals Scientific Procedures Act 1986 (ASPA).

## Author contributions

JA, JP, LD, EO, IVA, KWN, NF, and WB performed the experiments. JA, JP, AK, EO, GRY, KSSE, IVA, KWN, NF, WB, and GK analyzed the data. VP, CS, and GK supervised the study. JA, JP, and GK wrote the manuscript.

## Supplementary Material

Supplemental data

Supporting data values

## Version 1 05/16/2023

In-Press Preview

## Version 2 07/17/2023

Electronic publication
