# Supplementary material for: Human endogenous retrovirus onco-exaptation counters cancer cell senescence through calbindin
Source: J Clin Invest. 2023 Jul 17;133(14):e164397. doi: 10.1172/JCI164397 (PMC10348765; doi:10.1172/JCI164397)

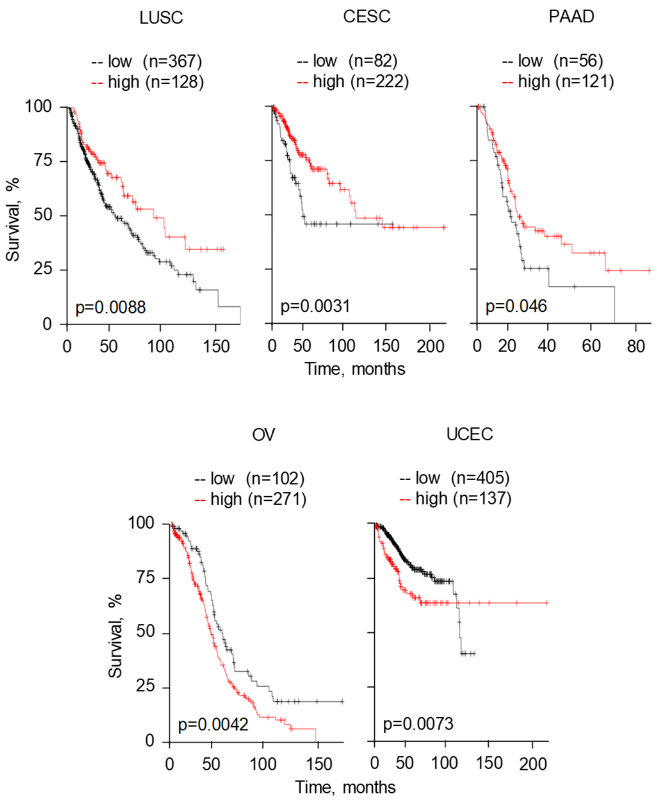

Supplemental Figure 1. Association between *CALB1* expression and overall survival of distinct cancer types. Overall survival of patients with indicated cancer types stratified according to expression of CALB1. Data are from TCGA, plotted using the Kaplan-Meier Plotter web-based survival analysis tool (http://kmplot.com).

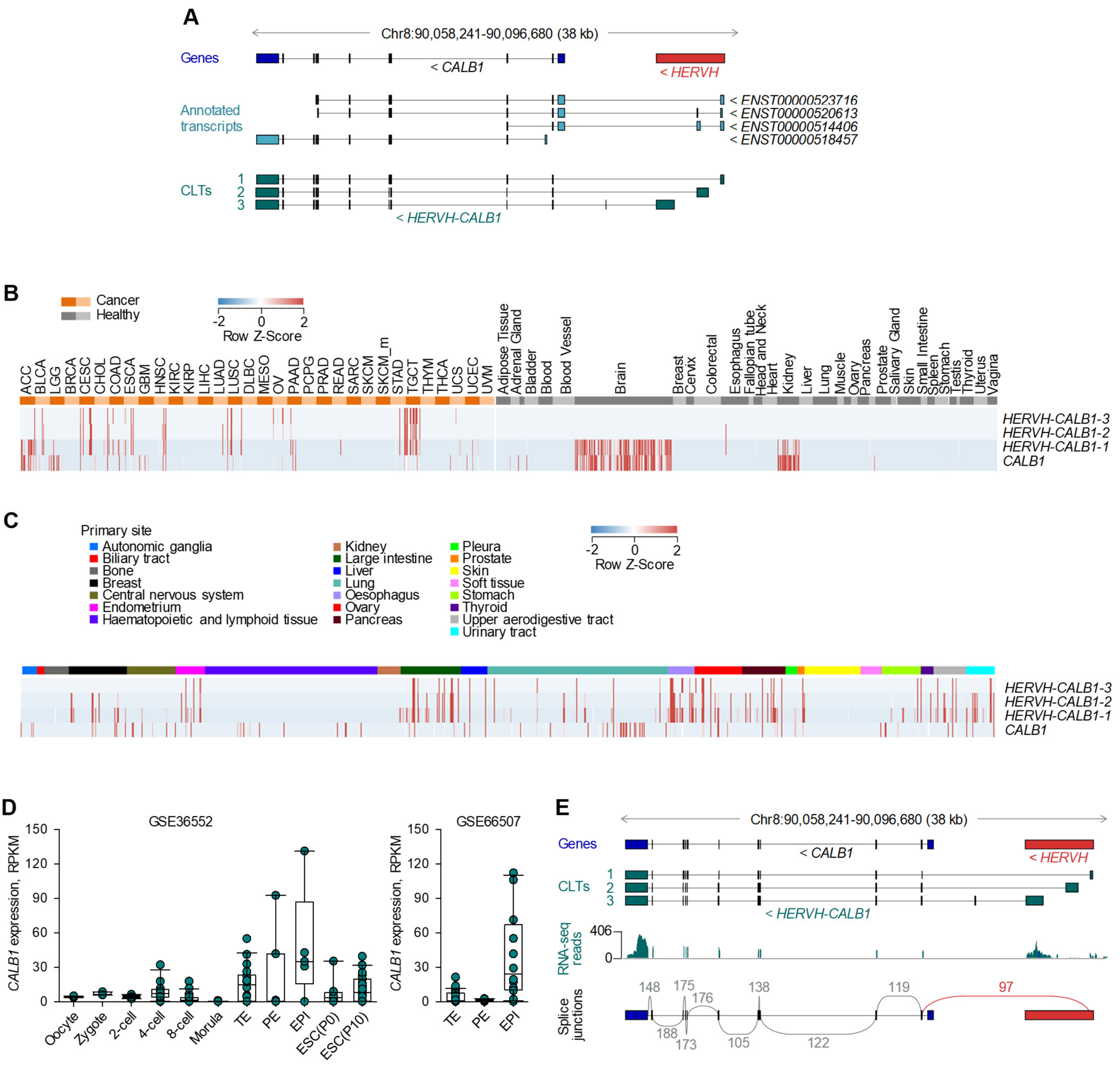

Supplemental Figure 2. Expression of *CALB1* and *HERVH-CALB1* isoforms in cancer and healthy tissues. (**A**)
The canonical GENCODE annotated transcript (Gene), the integrated HERVH provirus, additional annotated transcripts and assembled CLTs at the CALB1 locus. (**B**) Expression of the indicated isoforms in distinct TCGA cancer types and respective healthy tissues. Cancer types are each represented by 24 samples from TCGA, whereas healthy tissues are represented by a mixture of TCGA and GTEx samples (n=2-156) as described previously (Attig et al., 2019). (**C**) Expression of the indicated isoforms in cancer cell lines from CCLE grouped according to their histotype. (**D**) Expression of *CALB1* in the indicated stages of human embryogenesis from analysis of scRNA-seq datasets GSE36552 and GSE66507. EPI, epiblast; PE, primitive endoderm; TE, trophectoderm; ESC, ES cell; P, passage. (**E**) RNA-seq trace and number of splice junctions at the *CALB1* locus, determined by analysis of scRNA-seq data from the sample SRR491022 (late blastocyst, epiblast in dataset GSE36552). The canonical GENCODE annotated transcript, the integrated HERVH provirus and assembled CLTs are also shown for reference.

EF-hand   Calcium binding   Interaction with RANBP9 and IMPase

```
Calbindin      1 MAESHLQSSLITASQFFEIWLHFDADGSGYLEGKELQNLIQELQQARKKAGLELSPEMKTFVDQYGQRDDGKIGIVELAHVLPTEENFL 89
HERVH-CALB1    1 ---------------------------------------------------------MKTFVDQYGQRDDGKIGIVELAHVLPTEENFL 32

Calbindin     90 LLFRCQQLKSCEEFMKTWRKYDTDHSGFIETEELKNFLKDLLEKANKTVDDTKLAEYTDLMLKLFDSNNDGKLELTEMARLLPVQENFL 178
HERVH-CALB1   90 LLFRCQQLKSCEEFMKTWRKYDTDHSGFIETEELKNFLKDLLEKANKTVDDTKLAEYTDLMLKLFDSNNDGKLELTEMARLLPVQENFL 121

Calbindin    179 LKFQGIKMCGKEFNKAFELYDQDGNGYIDENELDALLKDLCEKNKQDLDINNITTYKKNIMALSDGGKLYRTDLALILCAGDN 261
HERVH-CALB1  179 LKFQGIKMCGKEFNKAFELYDQDGNGYIDENELDALLKDLCEKNKQDLDINNITTYKKNIMALSDGGKLYRTDLALILCAGDN 204
```

Supplemental Figure 3. Comparison of canonical and *HERVH-CALB1*-encoded Calbindin. Amino acid sequence alignment of the canonical Calbindin isoform (UniProt ID: P05937-1) and the translation product of the *HERVH-CALB1* transcript. The latter matches an annotated isoform with UniProt ID: P05937-2, but the corresponding annotated transcript ENST00000518457 (CALB1-208), is unlikely to be the source. The active Ca^{2+}-binding domains are indicated in red. The main domain of interaction with RAN Binding Protein 9 (RANBP9) and Inositol monophosphatase (IMPase) is indicated in green.

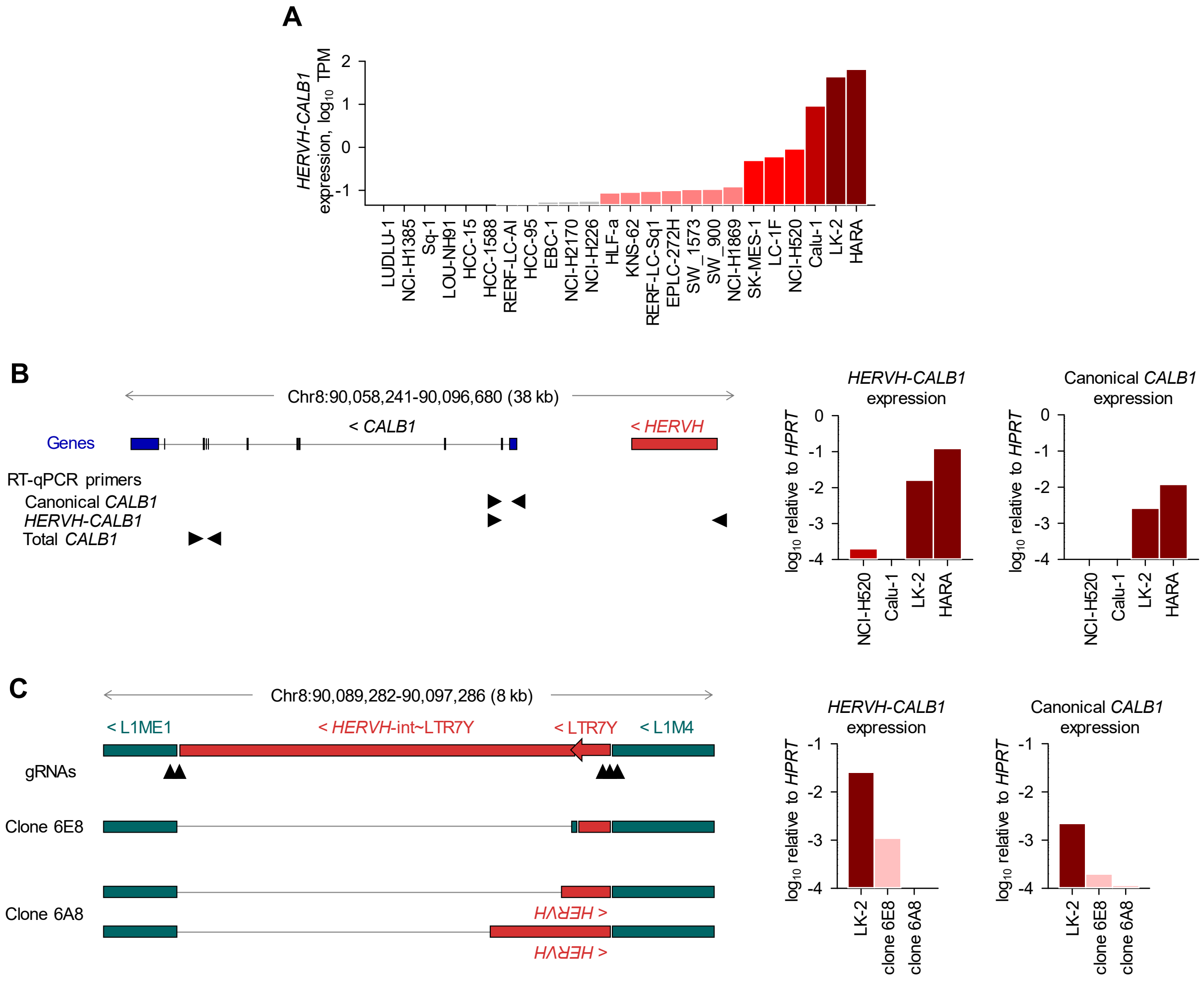

Supplemental Figure 4. ***HERVH*-dependent expression of CLAB1 in LUSC cell lines.** (**A**) *HERVH-CALB1* expression, assessed by analysis of RNA-seq data, in the indicated LUSC cell lines from CCLE. (**B**) Diagram depicting the location of PCR primers (black arrows, pointing the end position of each primer) used for the amplification of the canonical *CALB1* transcript (exons 1 and 2), the *HERVH-CALB1* transcript (*HERVH* and exon 2) or both (exons 7-10) (*left*), and expression of the canonical *CALB1* or *HERVH-CALB1* transcripts, relative to expression of *HPRT*, determined by RT-qPCR in the indicated LUSC cell lines (*right*). (**C**) Diagram depicting the position of the gRNAs (black arrows) used by Cas9-mediated deletion of the *HERVH* provirus upstream of the *CALB1* gene and resulting alleles in LK-2 clones 6E8 and 6A8 (*left*), and expression of the canonical *CALB1* or *HERVH-CALB1* transcripts, relative to expression of *HPRT*, determined by RT-qPCR in the same LK-2 clones and parental LK-2 cells (*right*). Clone 6E8 sustained a near complete deletion of the 5.4 kb *HERVH* provirus (with a residual 0.5 kb segment) and clone 6A8 showed extensive deletions with inversion of a residual 0.6 kb and 1.5 kb segments in separate alleles, respectively. In both clones, expression of the *HERVH-CALB1* transcript, which accounted for over 90% of total CALB1 transcription in these cells, was reduced by over 1 log.

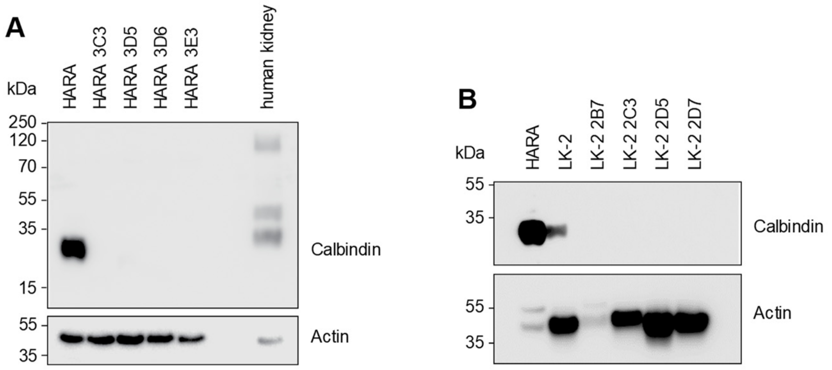

Supplemental Figure 5. Calbindin expression in *CALB1*-deficient HARA and LK-2 cells. (**A-B**) Western blots of Calbindin and Actin in lysates from parental HARA cells and *CALB1*-deficient HARA cell clones (A) and from parental LK-2 cells and *CALB1*-deficient LK-2 cell clones (B). Human kidney lysate is also included as control in (A) and HARA cells are included as a control in (B). HARA cell lysates are underloaded in (B) to compensate for higher expression of Calbindin in HARA than in LK-2 cells.

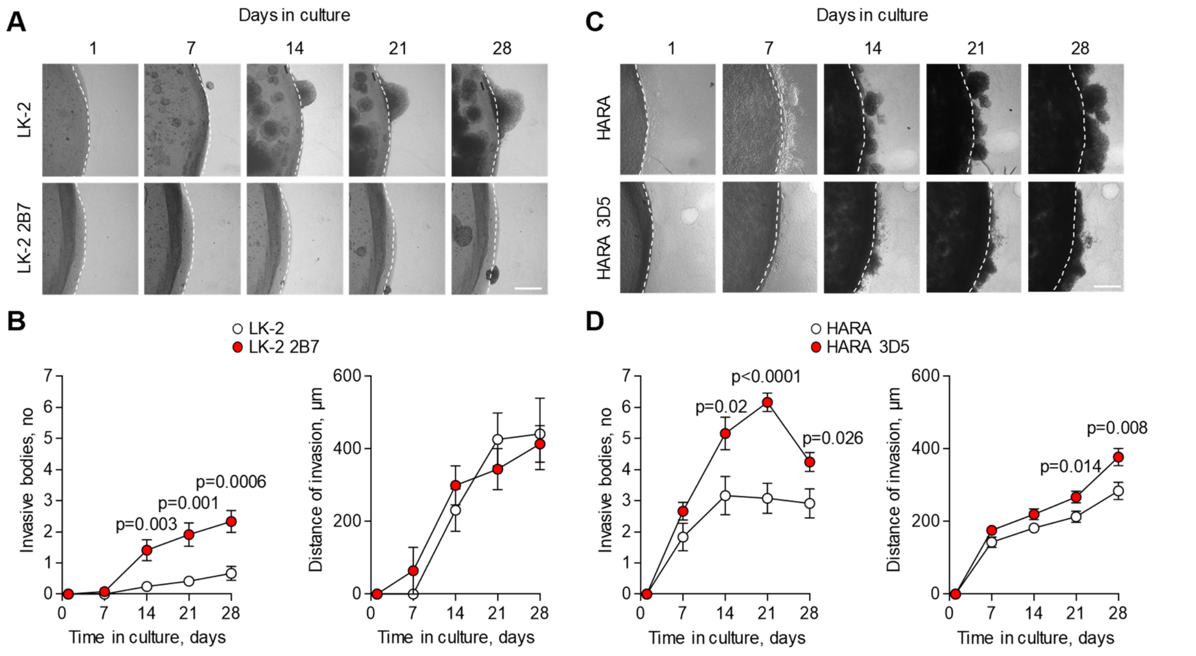

Supplemental Figure 6. Effect of Calbindin deficiency on cell growth in 3D collagen matrices. (**A**) Phase contrast images of LK-2 cell and of Calbindin-deficient LK-2 2B7 cell 3D collagen matrix cultures over time. (**B**) Mean number of invasion bodies (±SEM) in the seeded collagen matrices (n=12) (*left*) and mean (±SEM, n=3-14 measurements per time-point) distance (*right*) of LK-2 and LK-2 2B7 cell invasion into the stromal compartment. (**C**) Phase contrast images of HARA cell and of Calbindin-deficient HARA 3D5 cell 3D collagen matrix cultures over time. (**D**) Mean number of invasion bodies (±SEM) in the seeded collagen matrices (n=12) (*left*) and mean (±SEM, n=13-42 measurements per time-point) distance (*right*) of HARA and HARA 3D5 cell invasion into the stromal compartment. In (A) and (C), dashed lines denote the boundaries of the artificial cancer masses (scale bar=500 µm). P values shown are uncorrected for multiple comparisons.

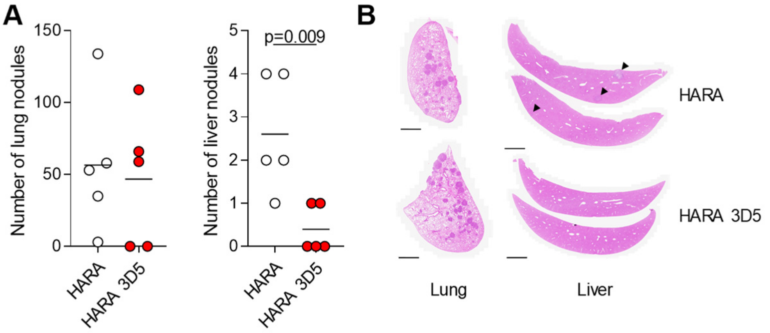

Supplemental Figure 7. HARA cell growth in immunodeficient recipient mice. (**A**) Number of lung nodules in the lungs (*left*) or livers (*right*) of immunodeficient *Rag2*$^{-/-}$*Il2rg*$^{-/-}$ *Cd47*$^{-/-}$ recipient mice following intravenous injection of HARA cells or Calbindin-deficient HARA 3D5 cells. (**B**) Haemotoxylin and Eosin staining of lungs and livers from the same groups of recipient mice. In liver sections, the black arrows indicate tumor nodules. The lung and liver sections are from the same recipient mouse in each group (scale bars=2 mm and =0.5 mm for lungs and livers, respectively). The data shown are from a single experiment.

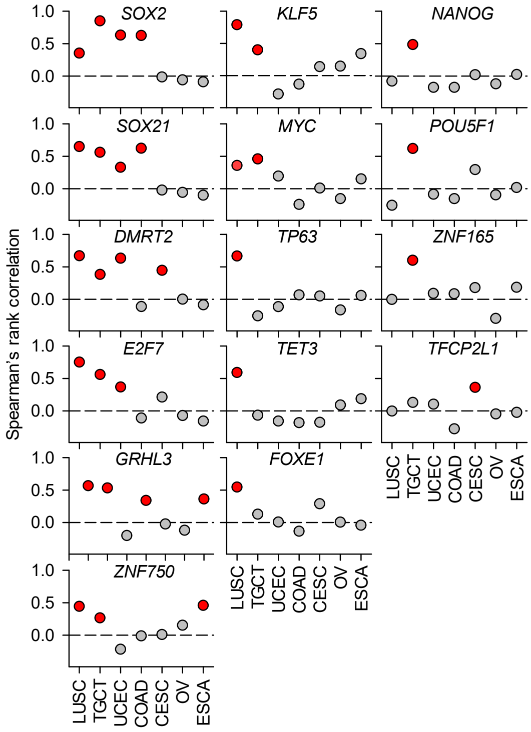

Supplemental Figure 8. Correlation of *HERVH-CALB1* and major transcription factor expression in distinct cancer types. Spearman's rank correlation of *HERVH-CALB1* expression and major transcription factor expression in in RNA-seq data from the indicated TCGA cancer types (n=24 per cancer type).

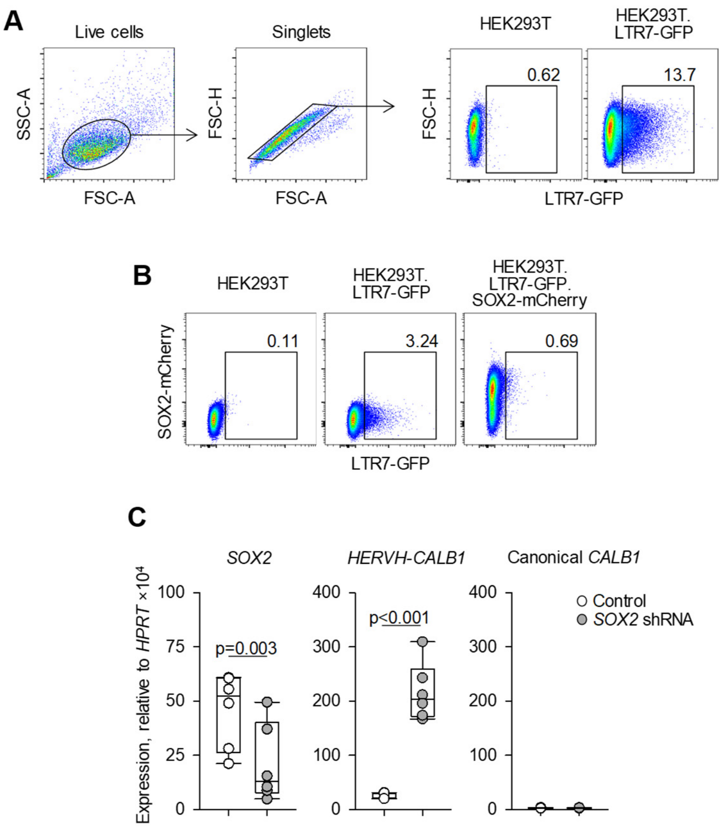

Supplemental Figure 9. Effect of SOX2 on *HERVH* LTR7 activity. (**A**) Flow cytometric example depicting the gating strategy for the identification of HEK239T cells transduced to express the *HERVH* LTR7-GFP reporter (HEK239T.LTR7-GFP cells). (**B**) *HERVH* LTR7-GFP expression in HEK293T cells, HEK239T.LTR7-GFP cells and in HEK239T.LTR7-GFP cells additionally transduced to express SOX2 and mCherry from a bicistronic construct. **c,** Transcription of SOX2 and of the *HERVH-CALB1* and canonical *CALB1* transcripts, relative to expression of *HPRT*, determined by RT-qPCR in SW620 cells and SW620 cells in which *SOX2* was knocked down by shRNA. Data are pooled from 2 independent experiments with 3 replicates per experiment.

A

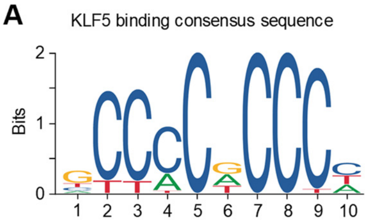

B

Preferred bases in all positions
Second preference in one position

```
>LTR7 DF0000571.4
TGTCAGGCCTCTGAGCCCAAGCTAAGCCATCGCATCCCCTGTGACCTGCACGTACACGTCCAGATGGCCTGAAGTAACTGAAGA
ATCACAAAGAAGTGAANATGCCCTGCCCCGCCTTAACTGATGACATTCCACCACAAAAGAAGTGAAAATGGCCNGTCCCTGCCT
TAACTGATGACATTACCTTGTGAAATTCCTTCTCCTGGCTCATCCTGGCTCAAAAAGCTCCCCCACTGAGCACCTTGCGACCCC
CACTCCTGCCCGCCAGAGAACAACCCCCCTTTGACTGTAATTTTCCTTTACCTACCCAAATCCTATAAAACGGCCCCACCCCTA
TCTCCCTTCGCTGACTCTCTTTTCGGACTCAGCCCGCCTGCACCCAGGTGATAAACAGCCTTGTTGCTCACACAAAGCCTGTTT
GGTGGTCTCTTCACACGGACGCGCGTGACA

>LTR7-GFP reporter
TGTCAGGCCTCTGAGCCCAAGCCAAGCCATCGCATCCCCTATGACATGCACGTACACGCCCAGATGGCCTGAAGTAACTGAAGA
ATCACAAAAGAAGTGAATATGCCCTGCCCCACCTTAACTGATGACATTCCACCACAAAAGAAGTGTAAATGGCCAGTCCTTGCC
TTAACTGATGACATTACCTTGTGAAAGTCCTTTTCCTGGCTCATCCTGGCTCAAAAAGCACCCCCACTGAGCACCTTGCGACCC
CCCGCTCCTACCCGCCAGAGAACAAACCCCCTTTGACTGTAATTTTCCTTTACCTAACCAAATCCTATAAAACGGCCCCACCCT
TATCTCCCTTCGCTGACTCTCTTTTCGGACTCAGCCCGCCTGCACCCAGGTGAAATAAACAGCCTCGTTGCTCACACAAAGCCT
GTTTGGTGGTCTCTTCACACGGACGCGCATGAAA

>LTR7B DF0000587.4
TGTCAGGCCTCTGAGCCCAAGCTAAGCCATCATATCCCCTGTGACCTGCACGTATACATCCAGATGGCCTGAAGCAACTGAAGA
TCCACAAAAGAAGTGAAAATAGCCTTAACTGATGACATTCCACCATTGTGATTTGTTTCTGCCCCACCCTAACTGATCAATGTA
CTTTGTAATCTCCCCCACCCTTAAGAAGGTNCTTTGTAATCTCCCCCACCCTTAAGAAGGTTCTTTGTAATTCTCCCCACCCTT
GAGAATGTACTTTGTGAGATCCACCCCCTGCCCGCAAAACATTGCTCCTAACTCCACCGCCTATCCCAAAACCTATAAGAACTA
ATGATAATCCCACCACCCTTTGCTGACTCTCTTTTCGGACTCAGCCCGCCTGCACCCAGGTGAAATAAACAGCCTTGTTGCTCA
CACAAAGCCTGTTTGGTGGTCTCTTCACACGGACGCGCGTGACA

>LTR7C DF0000588.4
TGTCAGGCCTCTGAGCCCAAGCTAAGCCATCGTAACCCCTGTGACCTGCACGTATACATCCAGATGGCCTGCGGGNNNCAANNN
NNNCGGAGCAACCGAAGAACCACAAAAGAAGTGAAACAGCCAGTTCCTGCCTTAACTGATTNACCGACGTTACGACATTCCACC
ATTGTGATTTGTTCCTGCCCCACCCTAACTGATCAATCGACCTTGTGACATTCTTCTCCTGGACAATGAGTCTCATGATCTCCC
CACCGNGCACCTTGTGACCCCCGCCCCTGCCCGCAAGAGATAACCACCTTTAACTGTAATTTTCCACTACCTACCCAAATCCTA
TAAAACTGCCCCACCCCTATCTCCCTTCGCTGACTCCCTTTTCGGACTCAGCCCGCCTGCACCCAGGTGAATAAACAGCCTTGT
TGCTCACACAAAGCCTGTTTNGGTGGTCTCTTCACACGGACGCGCGTGACA

>LTR7Y DF0000589.4
TGTCAGGCCTCTGAGCCCAAGCCAAGCCATCGCATCCCCTGTGACTTGCACGTATACGCCCAGATGGCCTGAAGTAACTGAAGA
NCCACAAAAGAAGTGAAAACNGCCTTAACTGATGACATTCCACCATTGTGATTTGTTCCTGCCCCACCCTAACTGAGCGATNNA
CCCTGTAATCTCCCCCNCCCTTNAGAAGGTNNNNCGNCGNCTCCCCCACCCCCGAGAANNNTCTTTGTAATTCTCCCCACCCTT
GAGAATGCACCTTGTGACCCCCGCCCCTGCCCACCAGAGAACAACCCCCTTTGACTGTAATTTTCCATTACCTTCCCAAATCCT
ATAAAACGGCCCCACCCCTATCTCCCTTCGCTGACTCTCTTTTCGGACTCAGCCCGCCTGCACCCAGGTGAAATAAACAGCCAT
GTTGCTCACACAAAGCCTGTTTGGTGGTCTCTTCACACGGACGCGCATGAAA

>LTR7Y HERVH-CALB1 5’
TGTCAGGCCTCTGAGCCCAAGCCAAGCCATCACATCCCCTGTGACTTGCACGTATACACCCAGATGGCCTGAAGTAACTGGAGA
ATCACAAAAGAAGTGAAAAGGCCCTGCCCCGCCTTAACTGATGACATTCCACCATTGTGATTTGTTCCTGCCCCACCTTAACTG
AGTGATTAACCCTGTGAATTTCCTTGTCCTGGCTCAGAGGCTCCCCCACTGAGCACCTTGTGACCACTGCCCCTGCCCACCAGA
GAACAACCCCCTTTGGCTGTAATTTTCCATTACCTTTCCAAATCCTATAAAACGGCCCCACCCCTATCTCCCTTCGCTGACTCT
CTTTTTGGACTCAGCCCACCTGCACCCAGGTGAAATAAACAGCCATGTTGCTCACACAAAGCCTGTTTGGTGGTCTCTTCACAT
GGACGCACATGAAA

>LTR7Y HERVH-CALB1 3’
TGTCAGGCCTCTGAGCCCAAGCCAAGCCATCACATCCCCTGTGACTTGCACGTATACACCCAGATGGCCTGAAGTAACTGGAGA
ATCACAAAAGAAGTGAAAAGGCCCTGCCCCGCCTTAACTGATGACATTCCACCATTGTGATTTGTTCCTGCCCCACCTTAACTG
AGTGATTAACCCTGTGAATTTCCTTGTCCTGGCTCAGAGTCTCCCCCACTGAGCACCTTGTGACCCCCGCCCCTGCCCACCAGA
GAACAACCCCCTTTGACTGTAATTTTCCATTTCCTTTCCAAATCCTATAAAACGGCCCCACCCCTATCTCCCTTCGCTGACTCT
CTTTTTGGACTCAGCCCACCTGCACCCAGGTGAAATAAACAGCCATGTTGCTCACACAAAGCCTGTTTGGTGGTCTCTTCACAT
GGACGTGCATGAAA
```

Supplemental Figure 10. KLF5 binding sites present in *HERVH* LTRs. (**A**) KLF5 binding consensus sequence from (https://jaspar.genereg.net/matrix/MA0599.1). The negative strand is shown. (**B**) *HERVH* LTR consensus sequences and sequence of the LTR7 in the LTR7-GFP reporter, and of the 5’ and 3’ LTRs of the *CALB1*-associated *HERVH* provirus, with KLF5 binding sites highlighted. Red highlighting corresponds to a perfect KLF5 binding site (with preferred bases in all positions) and grey highlighting corresponds to an imperfect site (with the second preferred base in one position of the KLF5 binding motif).

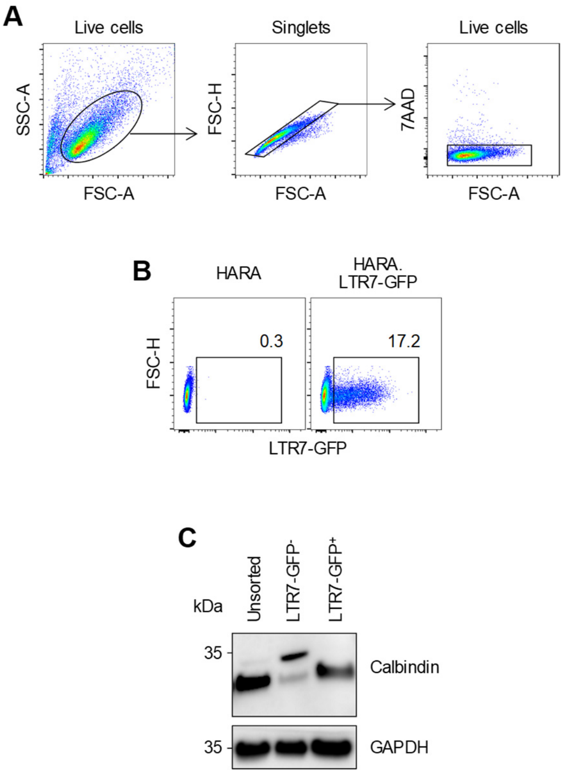

Supplemental Figure 11. *HERVH* LTR7-GFP reporter activity in HARA cells. (**A**) Flow cytometric example depicting the gating strategy for the identification of HARA cells transduced to express the *HERVH* LTR7-GFP reporter (HARA.LTR7-GFP cells). (**B**) *HERVH* LTR7-GFP expression in HARA and HARA.LTR7-GFP cells. (**C**) Western blots of Calbindin and GAPDH in lysates from unsorted HARA.LTR7-GFP cells and from FACS-purified HARA.LTR7-GFP$^-$ and HARA.LTR7-GFP$^+$ cells. The higher and lower molecular Calbindin isoforms correspond to the canonical and *HERVH-CALB1*-encoded Calbindin, respectively.

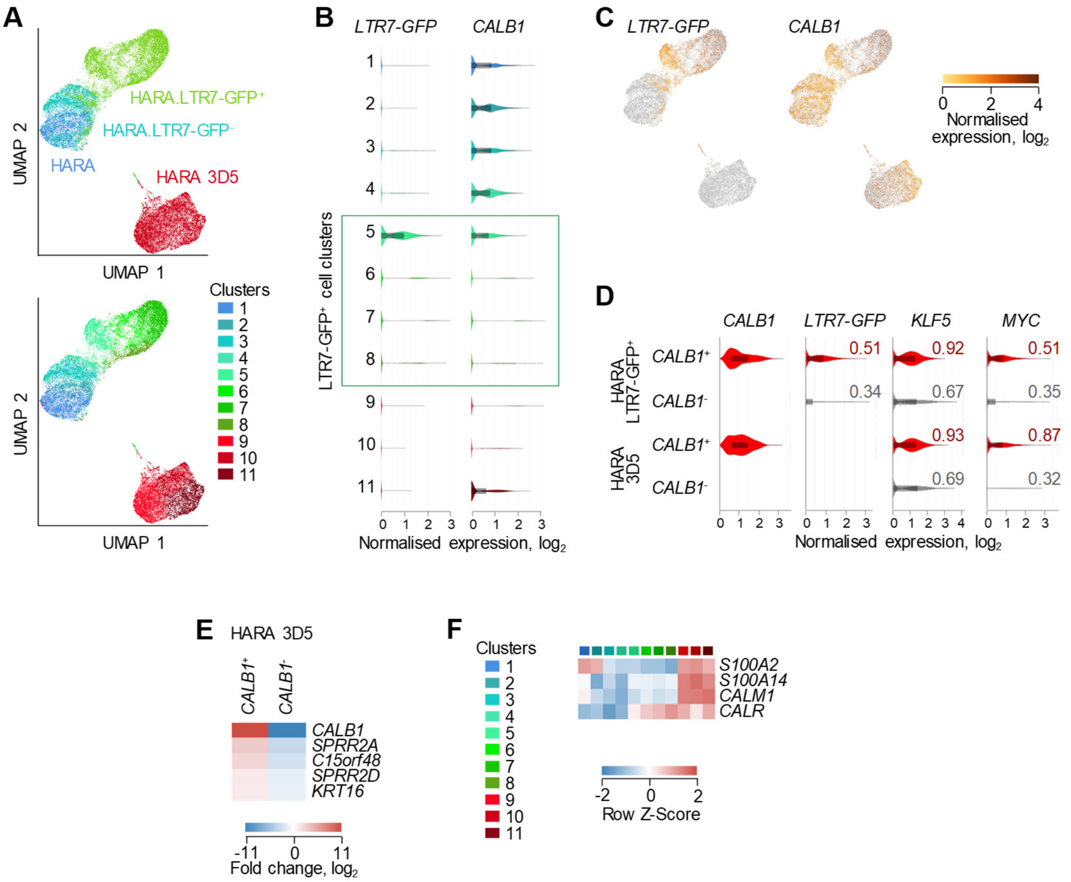

Supplemental Figure 12. Analysis of scRNA-seq data from HARA cells according to *CALB1* genotype and *HERVH* LTR7 reporter activity. (**A**) UMAP clustering of HARA cells, Calbindin-deficient HARA 3D5 cells, HARA.LTR7-GFP^{+} and HARA.LTR7-GFP^{-} cells according to scRNA-seq profiling. Cells are labelled by their genotype/phenotype (*top*) or by the cluster they were assigned (*bottom*). (**B**) Violin plots of normalized expression of *HERVH* LTR7-GFP reporter and *CALB1* in the indicated cell clusters in (A). Boxed are the clusters (5-8) containing the HARA.LTR7-GFP^{+} cells. (**C**) Normalized expression of *HERVH* LTR7-GFP reporter and *CALB1* in UMAP cell cluster projections as in (A). (**D**) Violin plots of normalized expression of *CALB1*, *LTR7-GFP* reporter, *KLF5* and *MYC* in HARA.LTR7-GFP^{+} and HARA 3D5 cells, separated based on *CALB1* mRNA expression. (**E**) Heatmap of fold-change of the indicated genes differentially expressed ($p<0.05$) between HARA 3D5 cells separated based on *CALB1* mRNA expression. (**F**) Heatmap of expression of genes encoding the indicated calcium-regulated proteins in different cell clusters defined in (A).

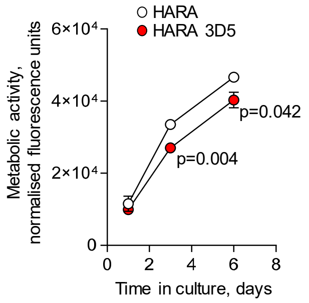

Supplemental Figure 13. Metabolic activity of HARA and HARA 3D5 cells. Mitochondrial metabolic activity of HARA and Calbindin-deficient HARA 3D5 cells over time, measured with the PrestoBlue Cell Viability Assay. Symbols are the mean (±SEM) of triplicate cultures, each measured in triplicate (p values calculated with Student's t-test). One representative of three independent experiments is shown.

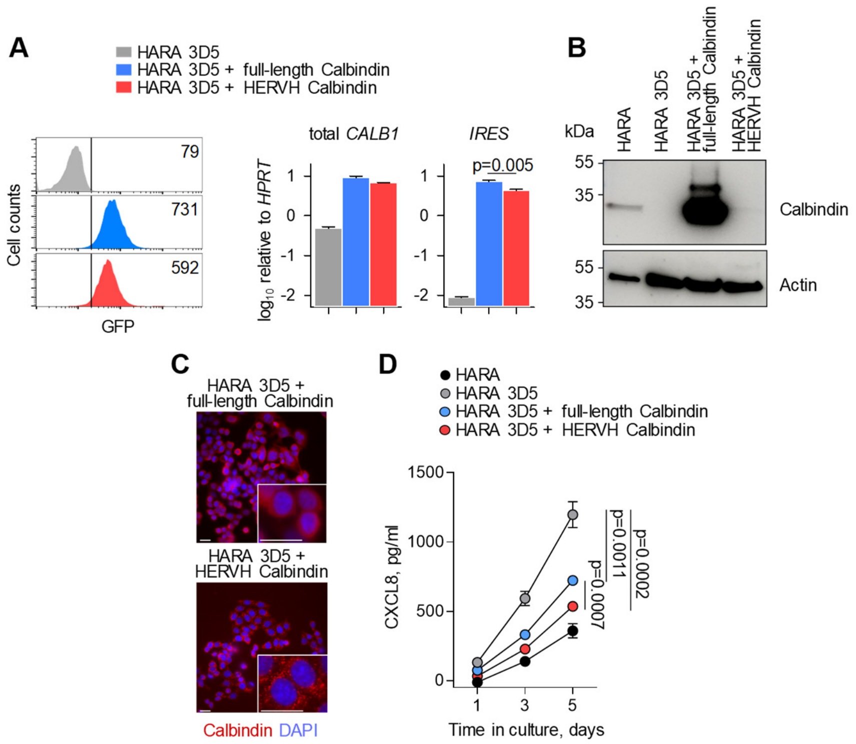

Supplemental Figure 14. Comparison of Calbindin isoform activity. (**A**) (*Left*) Flow cytometric detection of GFP expression in *CALB1*-deficient HARA 35D cells transduced to express the canonical full-length Calbindin or the *HERVH*-driven Calbindin isoform (HERVH Calbindin), each followed by IRES-driven GFP. Numbers within the plots denote the median fluorescence intensity of GFP. (*Right*) Expression of total *CALB1* and of the *IRES* sequence, relative to expression of *HPRT*, determined by RT-qPCR in the same cells. Values represent the mean (±SEM) of triplicates (p value calculated with Student's t-test). (**B**) Western blots of Calbindin and Actin in lysates from the same cells as in (A). The polyclonal antibody used for Calbindin detection underestimates the abundance of the *HERVH*-driven Calbindin isoform due its N-terminal truncation (Methods). (**C**) Immunofluorescence detection of Calbindin in HARA 35D cells transduced to express the full-length Calbindin or the *HERVH*-driven Calbindin isoform. Insets represent 4× magnification of the same images (scale bars=20 μm). Calbindin is detected here in red to avoid overlap with GFP expression in the cells. (**D**) Mean CXCL8 concentration (±SEM) over time in the supernatants of the same cells as in (A), determined by ELISA (n=3 per time-point) (p values calculated with Student's t-tests).

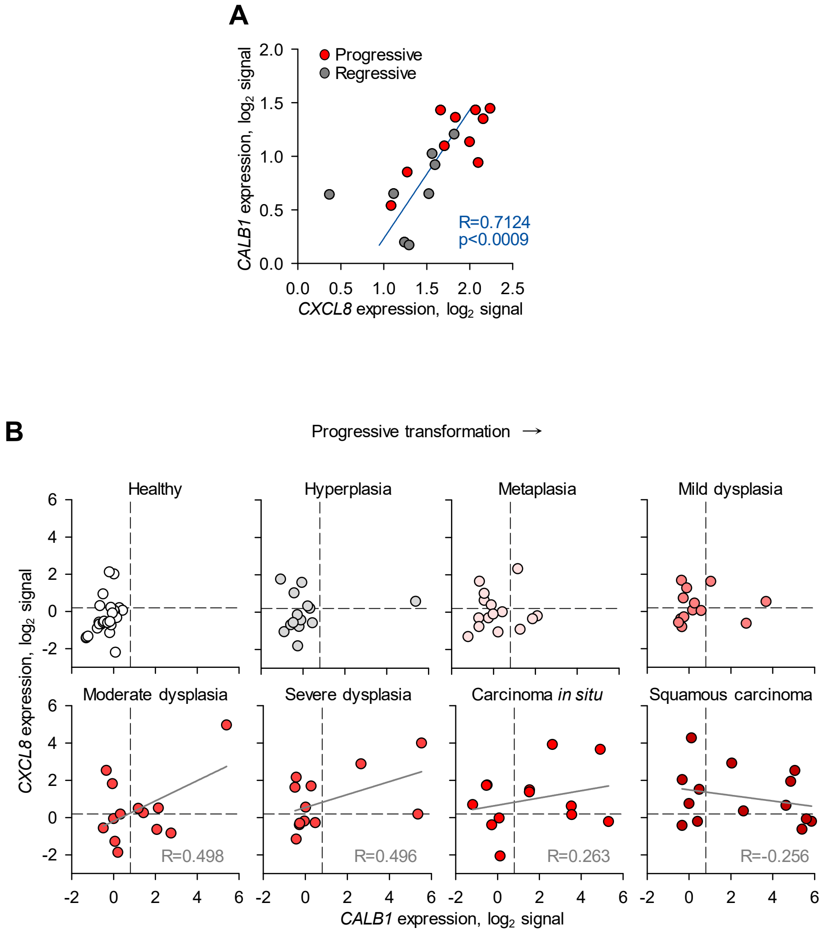

Supplemental Figure 15. Correlation of *CALB1* and *CXCL8* expression during progressive transformation to LUSC. (**A**) *CALB1* and *CXCL8* expression in microarray data (GSE108082) from precancerous lesions that progressed to LUSC (progressive) or spontaneously regressed (regressive). (**B**) *CALB1* and *CXCL8* expression in microarray data (GSE33479) from healthy lung tissue and from the indicated progressive stages preceding LUSC development. Also shown are regression lines for the later stages of progressive transformation. The correlation at these stages was not statistically significant ($p>0.05$). In (A) and (B), each symbol represents an individual patient.

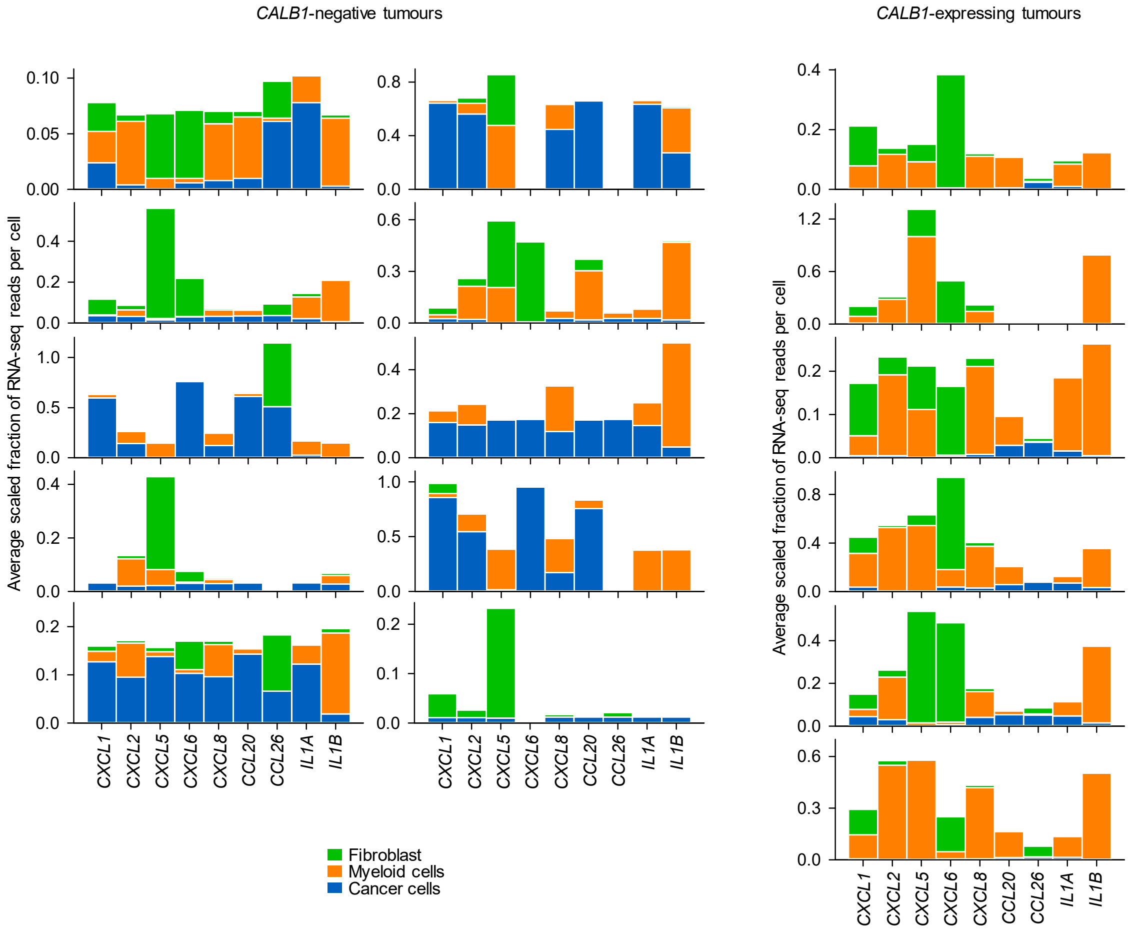

Supplemental Figure 16. Single-cell level source of chemokine and cytokine production in LUSC. Relative transcription of the indicated chemokines and cytokines in cancer cells, myeloid cells or tumor-associated fibroblasts, determined by analysis of scRNA-seq data of LUSC biopsies (GSE148071). For each chemokine/cytokine gene, the average fraction of RNA-seq reads found in each cell type is plotted, scaled to the relative number of cells of each type in each tumor biopsy, a according to CALB1 expression in cancer cells.

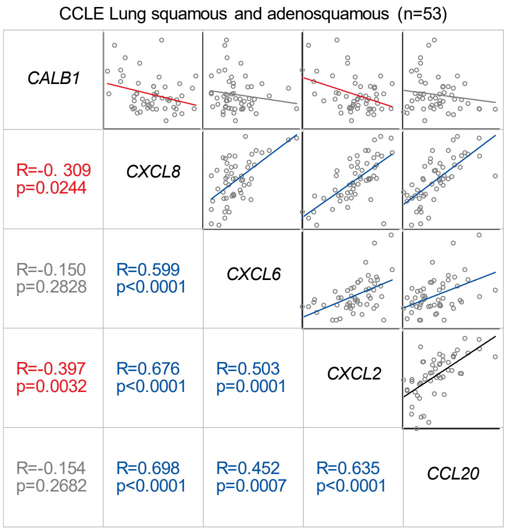

Supplemental Figure 17. *HERVH-CALB1* expression correlates with cancer cell-intrinsic chemokine production. Correlation between expression of *CALB1* and of the indicated chemokine/cytokine, in RNA-seq from lung squamous or adenosquamous cell lines from CCLE (n=53). Blue and red colors indicate significant positive and negative correlation, respectively, and grey color indicates lack of significant correlation.

Supplemental Table 1. HERVH-overlapping transcripts distinguishing Cluster 3 of LUSC samples.

transcript ID	chromosome	start	end	strand	length	LTR/repClass	LTR/repName	repClass	annotation
transae1d92c359843302	chr1	68386037	68391985	*	394	ERV1	HERVH-int~LTR7Y	LTR	unannotated
transc7fa2f3735e84ff3	chr1	68386093	68387300	*	1025	ERV1	HERVH-int~LTR7Y	LTR	unannotated
transdc91a198f74994c8	chr1	68386251	68391833	*	3640	ERV1	HERVH-int~LTR7Y	LTR	unannotated
trans2f40c5c9f3e9385e	chr1	68386357	68387072	*	594	ERV1	HERVH-int~LTR7Y	LTR	unannotated
trans05691997201f9e76	chr1	68386401	68387391	*	741	ERV1	HERVH-int~LTR7Y	LTR	unannotated
trans600e869a2b3cd998	chr1	68386608	68387045	*	376	ERV1	HERVH-int~LTR7Y	LTR	unannotated
trans62fce9061ceac841	chr1	68386990	68387312	*	259	ERV1	HERVH-int~LTR7Y	LTR	unannotated
trans3ca6c2f462ef6aa1	chr1	68387132	68388084	*	825	ERV1	HERVH-int~LTR7Y	LTR	unannotated
trans800c54f69211e914	chr1	68387132	68388954	*	1759	ERV1	HERVH-int~LTR7Y	LTR	unannotated
trans78209c415157bb28	chr1	68387170	68388727	*	1494	ERV1	HERVH-int~LTR7Y	LTR	unannotated
trans3d79b83d9b97c145	chr1	68387234	68448846	*	2097	ERV1	HERVH-int~LTR7Y;LTR17	LTR;SINE	partial
transac4ad524d9809ed3	chr19	43328209	43328885	*	556	ERV1	HERVH-int~LTR7Y	LTR	unannotated
trans6fb7437be6535be0	chr4	103557890	103558595	*	581	ERV1	HERVH-int~LTR7Y	LTR	unannotated
transb24f8043a4652c45	chr4	182814656	182815228	*	573	ERV1	HERVH-int~LTR7Y	LTR	unannotated
trans51d71bef55092f83	chr4	182814870	182815488	*	436	ERV1	HERVH-int~LTR7Y	LTR	unannotated
transc17b774084d73d69	chr4	182815251	182816498	*	1187	ERV1	HERVH-int~LTR7Y	LTR	unannotated
trans727d93d7baf06690	chr5	136541844	136545053	*	3146	ERV1	HERVH-int;LTR7Y	LTR	unannotated
trans4e063e2ceedb27fd	chr8	90058610	90091580	-	3722	ERV1	HERVH-int~LTR7Y	LTR	partial
trans0517ea10635ff667	chr8	90059213	90094338	-	2608	ERV1	HERVH-int~LTR7Y	LTR	partial
transfc7e8cbef2abec56	chr8	90090761	90095472	-	2908	ERV1	HERVH-int~LTR7Y;LTR7Y	LTR	unannotated
trans4bcbb61a599c4975	chr8	90093872	90094998	*	1066	ERV1	HERVH-int~LTR7Y	LTR	unannotated
trans0349ca8411f5e8b6	chr8	90094530	90094827	*	256	ERV1	HERVH-int~LTR7Y	LTR	unannotated
transa2af6789a9a5c31a	chrX	3553216	3560161	*	358	ERV1	HERVH-int~LTR7Y;LTR7Y	LTR	unannotated

Supplemental methods

Single-cell RNA-seq data analyses. All four cell populations (HARA cells, Calbindin-deficient HARA 3D5 cells, HARA.LTR7-GFP^{+} and HARA.LTR7-GFP^{-} cells) were processed as one batch at the same time, with cell viability between 89% and 93%. Cells were dispersed into single cell droplets using the 10× Genomics Chromium platform, and RNA was isolated and reverse transcribed with the 10× 3'-mRNA Seq (v3) kit. For analysis, reads were demultiplexed and processed with the Cell Ranger standard pipeline for each individual sequencing lane to identify low quality cells and empty beads. Corrected barcodes of valid cells were collected from all sequencing lanes of a sample and all reads from valid cells were remapped with HISAT2 (81) (optional parameters *-p 8 -q -k 5*) against the GRCh38 genome assembly, with the LTR7-GFP reporter sequence appended. The error-corrected cellular and molecular barcode sequences were recovered using the *MergeBamAlignment* utility in Picard (v2.23.0), which includes removal of multiple mapping locations. Downstream data analyses and visualization were carried out with the 10× Genomics Loupe Browser v5.

Additional single-cell RNA-seq data analyses. Additional single-cell RNA-seq data from LUSC biopsies were obtained from the public study GSE148071 (65). Three LUSC samples (P19, P31 and P36) were excluded as they contained fewer than 500 cells. Read count tables were downloaded and imported into Qlucore Omics Explorer v3.8 (Qlucore, Lund, Sweden) for downstream expression analyses and visualization.

Survival analysis and hazard ratio calculations. For survival analysis, all TCGA LUSC samples with survival data recorded were used. To test if expression of a transcript of interest correlated with patients' survival, we identified the patients in the bottom and top percentile expression ("low" vs. "high" expression). Survival analysis was done using the survfit function of the survival R package (v2.42), using overall survival time based on days_to_death annotation, or the last update of the annotation in March 2017 (that is, 365 days*(2017 – year of birth) minus the age-at-diagnosis in days). To compare curves between low and high

expression tertiles, log-rank testing was used and a Cox regression model was built to test the assumption of proportional hazards holds. Hazard odd ratios are given based on the Cox regression model. Similarly, a Cox regression model was used to compare survival between multiple expression clusters. Kaplan-Meier plots were generated via the web-based survival analysis tool (http://kmplot.com) (82).

Functional gene annotation by gene ontology. Pathway analyses were performed using g:Profiler (https://biit.cs.ut.ee/gprofiler) with genes ordered by the degree of differential expression. P values were estimated by hypergeometric distribution tests and adjusted by multiple testing correction using the g:SCS (set counts and sizes) algorithm, integral to the g:Profiler server (83). For analysis of genes downregulated and upregulated in HARA 3D5 cells, a term size of 15 was selected as the minimum on g:Profiler. Senescence-associated genes were defined according to the Cell Senescence Gene Database (http://csgene.bioinfo-minzhao.org) (84) and by literature searches.

Protein structure prediction and visualization. Structures of the canonical and *HERVH-CALB1*-encoded Calbindin isoforms were modelled based on the solved structure of canonical Calbindin (Protein Data Bank ID: 6FIE) using the SWISS-MODEL protein structure homology-modelling server (https://swissmodel.expasy.org) and structures were visualised in 3D Molecule Viewer (Vector NTI).

Transfections and transductions. HEK293T and HARA cells carrying a *HERVH* LTR7-GFP reporter construct were generated with the Sleeping Beauty transposon system, using the Sleeping Beauty transposase SB100X and pT2 ESRG-LTR7-GFP plasmids (Addgene, Cat #34879 and Cat #62541, respectively), both gifts from Dr Zsuzsanna Izsvak, Max-Delbrück-Center for Molecular Medicine, Berlin, Germany. Cells were seeded in 6 cm plates one day before transfection with 1 µg of SB100X plasmid and 4 µg of pT2 ESRG-LTR7-GFP plasmid using Lipofectamin 3000 (Thermo Fisher Scientific). Three days after transfection, cells expressing low levels of GFP were sorted on an Avalon flow cytometer to enrich for clones with single integrations. HARA.LTR7-GFP cells were re-sorted for GFP$^+$ cells twice

more and populations of previously GFP^+ cells HARA.LTR7-GFP cells were then resorted for GFP^+ and GFP^- subpopulations. HEK293T.LTR7-GFP cells used for screening of transcription factors affecting *HERVH* LTR7-GFP reporter activity were sorted on the basis of low GFP expression (resulting from partial silencing of the random integrations of the LTR7-GFP transposon). HEK293T.LTR7-GFP cells were seeded in 6-well plates and transfected with 2 µg of plasmid each expressing the following transcription factors: KLF5 (pcDNA3.1-KLF5, Genewiz); KLF4 (pMXs-hKLF4; Addgene, Cat #17219); MYC (pMXs-hc-MYC; Addgene, Cat #17220) (both gifts from Prof Shinya Yamanaka, Center for iPS Cell Research and Application, Kyoto University, Kyoto, Japan); FOXE1 (pBABE FoxE1; Addgene, Cat #26350; a gift from Prof Matthew Meyerson, Dana-Farber Cancer Institute, Boston, MA, USA); SOX2 (pRV-HA-SOX2-IRES-mCherry, Genewiz); and SOX9 (pcDNA3.1-SOX9, Genewiz). GFP expression was assessed by flow cytometry 3 days after transfection.

HEK293T.LTR7-GFP cells were additionally transduced with a retroviral vector (pRV-HA-SOX2-IRES-mCherry) encoding SOX2 and mCherry fluorescent protein, where the two open reading frames were separated by an internal ribosome entry site (IRES). Retroviral particles used for transduction were prepared by co-transfection of HEK293T cells with plasmids encoding murine leukaemia virus (MLV) gag/pol (pHIT60), vesicular stomatitis virus (VSV) glycoprotein (pVSVg) and pRV-SOX2-IRES-mCherry, using GeneJuice transfection reagent (Novagen). HARA 3D5 cells were transduced with retroviral vectors encoding either the canonical full-length (pCCLsin PPT hPGK flCALB1-IRES-GFP) or truncated Calbindin (pCCLsin PPT hPGK sCALB1-IRES-GFP) and GFP, with the two open reading frames separated by an IRES. These vectors were based on a third-generation lentivirus vector (85), and were packaged by co-transfection of HEK293T cells with plasmids encoding human immunodeficiency virus (HIV) Gag-Pol (pMDL Gag-Pol), Rev (pRSV HIV Rev) and VSV glycoprotein (pMD2 VSVg), using GeneJuice transfection reagent (Novagen). Two days after transfection, culture supernatant was collected, filtered through a 0.45 µm filter and used to

transduce target cells. Transduction was achieved by centrifugation in the presence of polybrene (4 µg/mL) at 300 × *g* for 45 mins at room temperature.

For knock-down of *SOX2* expression, SW620 cells were transfected with a plasmid expressing *SOX2* shRNA (pLKO.1 Sox2 HM a; Addgene, Cat #26353; a gift from Matthew Meyerson, Dana-Farber Cancer Institute, Boston, MA, USA) and were selected with 1 µg/ml puromycin for 1 week prior to harvesting for RNA.

Cas9-mediated editing. The *HERVH* provirus in the *CALB1* locus was targeted by guide RNA (gRNA) sequences: 5'-GAATTTTCAAGAATTGTTAAT-3', 5'-GCCATTGTAGTCGATGTGTAG-3', 5'-GAGTCAATCAGATGCCTACTT-3', 5'-GTTAAATCTAAATGTATCTTA-3' and 5'-GCAAATGTTCTAATTTATCC-3'. For complete CALB1 loss of function, exon 4 of the *CALB1* gene was targeted by gRNA sequences 5'-GCTGGCATCGGAAGAGCAGC-3' and 5'-GTCCTGTGAGGAATTCATGA-3'. In all cases, gRNA sequences were synthesised by IDT with 35 nt overlaps and cloned into the pSpCa9(BB)-2A-GFP scaffold using NEBuilder® HiFi DNA Assembly (NEB; Cat #E5520). The pSpCa9(BB)-2A-GFP scaffold was a gift from Feng Zhang, Broad Institute of MIT and Harvard, Cambridge, MA, USA (Addgene, Cat #48138). Cas9 and gRNA plasmids were transfected into target cells using Lipofectamin 3000 (Thermo Fisher Scientific). HARA and LK-2 cells were cultured to confluence, passaged and seeded into 6 cm plates. The next day, the respective plasmids were resuspended with P3000 reagent in OptiMEM (2.5 µg plasmids + 5 µl P3000), mixed with 7.5 µl Lipofectamin 3000 and spread in droplets on adherent cells. One week later, cells were stained with 7AAD (Thermo Fisher Scientific) and sorted for live cells ($7AAD^-$) and intermediate expression of GFP. After recovery, single-cell suspensions were plated into 96-well plates at ~0.4 cells per well and single-cell clones were confirmed visually. After 4-6 weeks, genomic DNA samples were taken from all clones using QuickExtract DNA Extraction Solution (Epicentre). The *CALB1* locus was amplified with primers including Illumina adapter sequences, barcoded using the NEBNext Ultra II DNA Library kit and sequenced on a MiSeq sequencer (Illumina). After deconvolution, sequences were collapsed

by identity to the longest continuous sequence stretch and the frequency of each sequence was used to identify clones with frame-shift mutations on all alleles.

Cell growth and metabolism assays. Growth and proliferation of parental and *CALB1*-deficient HARA and LK-2 cells was assessed by real-time quantitative live-cell imaging using the Incucyte Live-Cell Analysis System (Sartorius). Additionally, the resazurin-based assay PrestoBlue Cell Viability Assay (Invitrogen, Cat #A13261) was used to measure the reducing efficiency of the same cells, which reports mitochondrial metabolic activity. Cells were seeded into 24-well plates at a density of 8×10^3 and 4×10^3 cells per well for HARA and LK-2 subclones, respectively. Measurements were taken at 24, 72 and 144 hours after seeding. The assays were performed according to manufacturer's instructions and read on the Spark multimode plate reader (TECAN). For the PrestoBlue fluorescence intensity measurements, supernatants were transferred to black 96-well plates (Männedorf, Switzerland) prior to reading.

3D collagen matrix invasion assay. Collagen matrices were constructed as previously described (86). Briefly, monomeric collagen type I of rat tail origin (First Link, Cat #60-30-810) was used at an initial 2.0 mg/ml concentration. The collagen was mixed with 10× MEM (Gibco, Cat #11430030) and a neutralizing agent composed of 17% 10 M NaOH (Sigma-Aldrich; #221465-25G) in 1 M HEPES (Sigma-Aldrich, Cat #15630080), according to the RAFT 3D Cell Culture Kit Protocol (https://bioscience.lonza.com). An initial tumor mass was created by mixing 5×10^5 cells/sample into the collagen mix and suspending 240 µl into a 96-well plate. The cellular collagen mix was left for 15 min at 37°C to crosslink and plastic compression was performed using the RAFT absorbers (Lonza, Cat #016-0R92 and Cat #016-1R32, for 96- and 24-well size respectively), increasing collagen density from 0.2% to 10%. This dense artificial tumor mass was then implanted into 1.3 ml acellular collagen mix, representing the stromal compartment, in a 24-well plate followed by the same incubation and plastic compression protocol creating a compartmentalized 3D tumor invasion model. The 3D samples were cultured for 28 days with a 50% medium change every 48 hours. Brightfield images were taken

every 7 days at the four cardinal points around the tumor-stroma interface on the EVOS^{TM} digital inverted microscope (Invitrogen, through Thermo Fisher Scientific).

In vivo tumor models. Mice received 3×10^5 LK-2 or LK-2 2B7 cells, or 10^6 HARA or HARA 3D5 cells intravenously in 100 µl PBS. To monitor tumor growth, some mice were imaged on a Perkin Elmer Quantum GX2 with a voxel size of 50 µm. Acquisition was done at high speed mode with a 4 min respiratory gating window at 90 kV, 88 µA, FOX 36 mm and images were reconstructed to 25 mm. Mice were culled when recipients of wild-type LK-2 or HARA cells had visible tumors by imaging in pilot experiments (6 weeks after injection) and at equivalent time points in subsequent experiment. All experiments were approved by the ethics committee of the Francis Crick Institute and conducted according to local guidelines and UK Home Office regulations under the Animals Scientific Procedures Act 1986 (ASPA).

Histology and immunocytochemistry. Tumor-bearing lungs and livers were fixed in 10% neutral buffered formalin (Sigma-Aldrich) for 24 hours and transferred to 70% ethanol. Fixed tissues were embedded in paraffin and 4 µm sections mounted on slides. Hematoxylin and eosin staining was performed using the automated Tissue-Tek Prisma slide stainer. For immunohistochemistry staining, consecutive sections were boiled in sodium citrate buffer (pH 6.0) for 15 min followed by incubation for 1 hour with anti-Calbindin (Atlas Antibodies, Cat #HPA023099) or p63 (Santa Cruz, Cat #sc-8343). Primary anti-Calbindin antibodies were detected using biotinylated goat anti-rabbit IgG antibodies (Vector, Cat #BA-1000-1.5), followed by Vector ABC HRP kit (Vector, Cat #PK-6100) and Vector DAB HRP kit (Vector, Cat #SK-4100). Slides were imaged using a Zeiss AxioScan slide scanner (Carl Zeiss Meditec AG). For immunofluorescent staining, cell monolayers grown on 1.5 coverglass dishes (MatTek, Cat #P35G-1.5-20-C) were fixed using 10% neutrally buffered formalin (Genta Medical) for 15 min. Permeabilization was carried out for 1 h at room temperature using 0.2% Triton X-100 and 1% bovine serum albumin (Sigma-Aldrich). Primary antibody incubation for Calbindin was performed overnight at 4°C using the anti-Calbindin antibody (Atlas Antibodies, Cat #HPA023099) at a 1:200 dilution. Similarly, primary antibody incubation for γH2AX was

performed overnight at 4°C using the anti-γH2AX antibody (Abcam, Cat. #ab81299) at a 1:200 concentration. Secondary antibody incubation using Goat Anti-Rabbit IgG H&L AlexaFluor488 Antibody (Abcam, Cat #ab150077) was carried out the next day for 2.5 hours at room temperature at a 1:1,000 dilution. F-actin was visualized using AlexaFluor568 phalloidin (Invitrogen, Cat #A22287) at 165 nM followed by 4′,6-diamidino-2-phenylindole (DAPI) counterstain, using NucBlue (Invitrogen, Cat #R37606) at 2 drops/ml for 15 min. Samples were imaged on the Zeiss Observer.Z1 (Carl Zeiss Meditec AG) using Micro-Manager 2.0 software. For enumerating γH2AX stained cells, cells exhibiting DNA damage were identified using a combination of morphological criteria (enlarged cell size and flattened cell shape) and distinctive γH2AX staining (strong pan-nuclear staining, chromatin reorganization and visible foci).

Anti-Calbindin antibody specificity. The anti-Calbindin antibody used here for immunocytochemistry, immunofluorescence and Western blotting (Atlas Antibodies, Cat #HPA023099) is a rabbit polyclonal antibody raised against an immunogen corresponding to amino acid residues 7-96 of the full-length canonical Calbindin. Owing to the N-terminal deletion of amino acid residues 1-57 in the *HERVH*-driven Calbindin isoform, the latter isoform overlaps with the immunogen by only 39 of a total of 90 amino acid residues, whereas the canonical Calbindin isoform overlaps completely. As a result, although reactive with both isoforms, this polyclonal antibody detects the canonical Calbindin isoform much more efficiently than the *HERVH*-driven Calbindin isoform.

Reverse transcriptase-based quantitative PCR (RT-qPCR). RNA was extracted from pelleted cells using QIAshredder columns (Qiagen) using the RNeasy kit (Qiagen), and cDNA was synthesized using the Maxima First Strand cDNA Synthesis Kit (Thermo Fisher Scientific). qPCR was performed using Applied Biosystems Fast SYBR Green (Thermo Fisher Scientific) with the primers specific for detection of the following transcripts: the canonical *CALB1* transcript (exons 1 and 2): forward primer 5’-CCACCTGCAGTCATCCCTC-3’; reverse primer 5’-ATCAAGTTCTGCAGCTCCTTTCC-3’; the *HERVH-CALB1* transcript (*HERVH* and exon 2):

forward primer 5’-AGCCCAAGAAACATCTCACCAA -3’; reverse primer 5’-CAGCCTTCTTTCGCGCCTG-3’; all *CALB1* transcripts (exons 7-10): forward primer 5’-CCAGGTTACTACCAGTGCAGG-3’; reverse primer 5’-TGTATCCATTGCCGTCCTGAT-3’; *HPRT* transcript: forward primer 5’-TGACACTGGCAAAACAATGCA-3’; reverse primer 5’-GGTCCTTTTCACCAGCAAGCT-3’. Expression of the transducing vectors encoding either the canonical full-length (pCCLsin PPT hPGK flCALB1-IRES-GFP) or truncated Calbindin (pCCLsin PPT hPGK sCALB1-IRES-GFP) and GFP was additionally compared using the following primers specific to the common IRES: forward primer 5’-TCGCCAAAGGAATGCAAGGT-3’; reverse primer 5’-GCCGCCTTTGCAGGTGTATC-3’. Values were normalized to *HPRT* expression using the ΔC_T method.

Western blotting. Cell lysates in RIPA buffer were lysed in SDS sample buffer and denatured for 15 mins at 95°C. Samples were run on a 4-20% gel (Bio-Rad), transferred to a PVDF membrane (Bio-Rad), and blocked in 5% (wt/vol) bovine serum albumin fraction V (Sigma-Aldrich) in TBS-T. Membranes were stained with the indicated primary antibodies and HRP-conjugated secondary antibodies to rabbit IgG (Cell Signaling Technology, Cat #7074; 1;1000 dilution) or mouse IgG (Cell Signaling Technology, Cat #7076; 1:1000 dilution), or with HRP-conjugated anti-actin antibodies (Abcam, Cat #ab49900; 1:25000). Blots were visualized by chemiluminescence on an Amersham Imager 600 (GE Healthcare).

Flow cytometry and cell sorting. Cell suspensions were run for GFP and mCherry detection on a LSR Fortessa (BD Biosciences) running BD FACSDiva v8.0 and analyzed with FlowJo v10 (Tree Star Inc.) analysis software. Cell sorting was performed using either a FACSAria Fusion cell sorter (Becton Dickinson) or an Avalon cell sorting system (Bio-Rad).

Neutrophil isolation, culture and time-lapse imaging. Neutrophils were isolated from peripheral human blood of consented healthy donors. Briefly, venous blood was collected in Heparin tubes, layered on Histopaque 1119 (Sigma-Aldrich) and centrifuged for 20 mins at 800 × *g*. The neutrophil layer was collected, washed and further purified by centrifugation for 20 mins at 800 × *g* on a discontinuous Percoll gradient (GE Healthcare), consisting of layers

with densities of 85%, 80%, 75%, 70% and 65%. Isolated neutrophils were seeded, 5×10^4 per well, in black 96-well plates (PerkinElmer), in HyClone Hank's Balanced Salt Solution (HBSS) with calcium and magnesium, without Phenol red (GE Healthcare), containing 10mM HEPES (Invitrogen) and 50% conditioned medium obtained from cultures of HARA and HARA 3D5 cells. Live cells were stained with 4 µg/ml Hoechst (membrane permeable; Thermo Scientific) and dead cells with 0.2 µM SYTOX green (membrane impermeable; Invitrogen). The cells were imaged on an inverted Nikon wide-field microscope system at 37°C and 5% CO_2. Four fields of view were acquired per well every 30 mins for 20-32 hours using a 40× objective. Quantification of the number of SYTOX positive cells over the total number cells was performed using an intensity-based thresholding mask (Otsu) on the SYTOX and Hoechst channel in Fiji. Half-live values represent the time-point at which 50% of the cells of the field of view were SYTOX positive.

ELISA. Cells were seeded into 24-well plates at a density of 2×10^5 cells/well and supernatant samples were collected at 24, 72 and 144 hours later. CXCL8 levels were measured by using the Human IL-8/CXCL8 Quantikine ELISA Kit (R&D Systems, Cat #D8000C), according to the manufacturer’s instructions. Resulting optical densities (ODs) were read on a Spark multimode plate reader (TECAN).

Supplemental methods references

81. Kim D, et al. Graph-based genome alignment and genotyping with HISAT2 and HISAT-genotype. *Nat Biotechnol.* 2019;37(8):907-915.
82. Lánczky A, Győrffy B. Web-Based Survival Analysis Tool Tailored for Medical Research (KMplot): Development and Implementation. *J Med Internet Res.* 2021;23(7):e27633.
83. Raudvere U, et al. g:Profiler: a web server for functional enrichment analysis and conversions of gene lists (2019 update). *Nucleic Acids Res.* 2019;47(W1):191-198.
84. Zhao M, et al. CSGene: a literature-based database for cell senescence genes and its application to identify critical cell aging pathways and associated diseases. *Cell Death Dis.* 2016;7(1):e2053.
85. Dull T, et al. A third-generation lentivirus vector with a conditional packaging system. *J Virol.* 1998;72(11):8463-8471.
86. Pape J, et al. Cancer invasion regulates vascular complexity in a three-dimensional biomimetic model. *Eur J Cancer.* 2019;119:179-193.

Full unedited gels for Supplemental Figure 5A-B

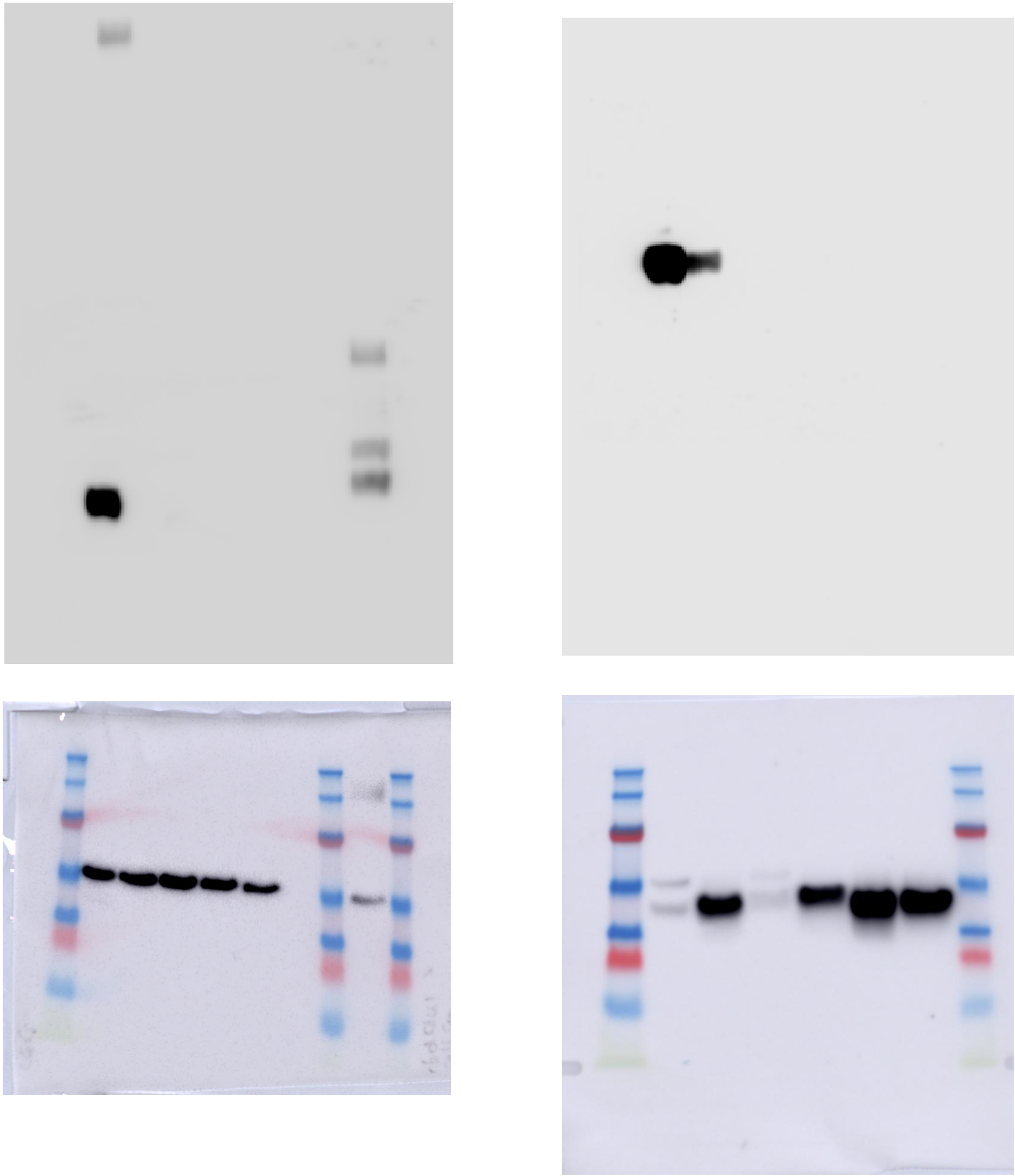

Full unedited gels for Supplemental Figure 11C

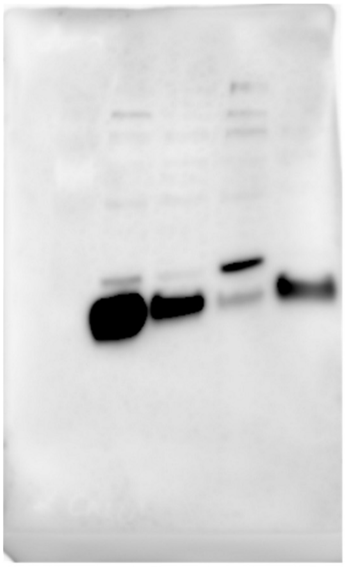

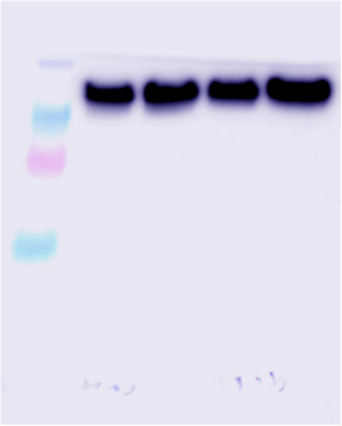

Full unedited gels for Supplemental Figure 14B

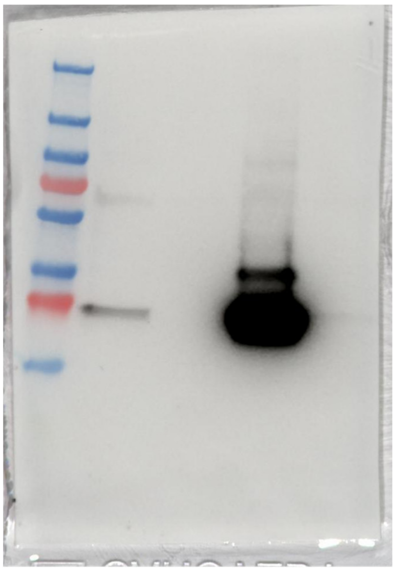

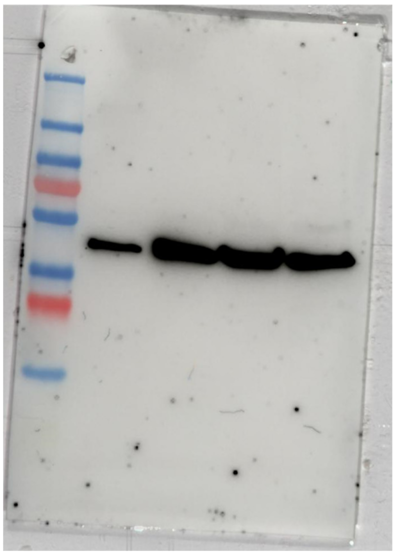

Supplement: Supplemental data [file jci-133-164397-s066.pdf]
